# Attenuated kidney oxidative metabolism in young adults with type 1 diabetes

**DOI:** 10.1172/JCI183984

**Published:** 2024-10-22

**Authors:** Ye Ji Choi, Gabriel Richard, Guanshi Zhang, Jeffrey B. Hodgin, Dawit S. Demeke, Yingbao Yang, Jennifer A. Schaub, Ian M. Tamayo, Bhupendra K. Gurung, Abhijit S. Naik, Viji Nair, Carissa Birznieks, Alexis MacDonald, Phoom Narongkiatikhun, Susan Gross, Lynette Driscoll, Maureen Flynn, Kalie Tommerdahl, Kristen J. Nadeau, Viral N. Shah, Tim Vigers, Janet K. Snell-Bergeon, Jessica Kendrick, Daniel H. van Raalte, Lu-Ping Li, Pottumarthi Prasad, Patricia Ladd, Bennett B. Chin, David Z. Cherney, Phillip J. McCown, Fadhl Alakwaa, Edgar A. Otto, Frank C. Brosius, Pierre Jean Saulnier, Victor G. Puelles, Jesse A. Goodrich, Kelly Street, Manjeri A. Venkatachalam, Aaron Ruiz, Ian H. de Boer, Robert G. Nelson, Laura Pyle, Denis P. Blondin, Kumar Sharma, Matthias Kretzler, Petter Bjornstad

**Affiliations:** 1 Department of Biostatistics and Informatics and; 2 Department of Pediatrics, Section of Endocrinology, University of Colorado School of Medicine, Aurora, Colorado, USA.; 3 Department of Medicine, Division of Neurology, Faculty of Medicine and Health Sciences, Université de Sherbrooke, Centre de Fecherche du Centre Hospitalier Universitaire de Sherbrooke (CRCHUS), Québec, Canada.; 4 Center for Renal Precision Medicine, University of Texas Health–San Antonio, San Antonio, Texas, USA.; 5 Department of Internal Medicine, Division of Nephrology, University of Michigan, Ann Arbor, Michigan, USA.; 6 Barbara Davis Center for Diabetes, University of Colorado Anschutz Medical Campus, Aurora, Colorado, USA.; 7 Diabetes Center, Department of Internal Medicine, Amsterdam University Medical Centers, location VUmc, Amsterdam, Netherlands.; 8 Radiology Department, Endeavor Health, Pritzker School of Medicine, University of Chicago, Chicago, Illinois, USA.; 9 Department of Radiology, University of Colorado School of Medicine, Aurora, Colorado, USA.; 10 Department of Medicine, Division of Nephrology, University of Toronto School of Medicine, Toronto, Ontario, Canada.; 11 Division of Nephrology, The University of Arizona College of Medicine Tucson, Tucson, Arizona, USA.; 12 University of Poitiers, INSERM, CHU Poitiers, Clinical Investigation Center CIC 1402, Poitiers, France.; 13 Department of Clinical Medicine, Aarhus University, Aarhus, Denmark.; 14 Department of Pathology, Aarhus University Hospital, Aarhus, Denmark.; 15 III. Department of Medicine, University Medical Center Hamburg–Eppendorf, Hamburg, Germany.; 16 Department of Population and Public Health Sciences and; 17 Department of Biostatistics, University of Southern California, Los Angeles, California, USA.; 18 Department of Pathology, The University of Texas Health San Antonio, San Antonio, Texas, USA.; 19 SygnaMap, Inc., San Antonio, Texas, USA.; 20 Kidney Research Institute, University of Washington, Seattle, Washington, USA.; 21 Chronic Kidney Disease Section, National Institute of Diabetes and Digestive and Kidney Diseases, Phoenix, Arizona, USA.; 22 Department of Medicine, Division of Metabolism, Endocrinology and Nutrition, University of Washington School of Medicine, Seattle, Washington, USA.

**Keywords:** Endocrinology, Metabolism, Diabetes

## Abstract

**BACKGROUND:**

In type 1 diabetes (T1D), impaired insulin sensitivity may contribute to the development of diabetic kidney disease (DKD) through alterations in kidney oxidative metabolism.

**METHODS:**

Young adults with T1D (*n* = 30) and healthy controls (HCs) (*n* = 20) underwent hyperinsulinemic-euglycemic clamp studies, MRI, ^11^C-acetate PET, kidney biopsies, single-cell RNA-Seq, and spatial metabolomics to assess this relationship.

**RESULTS:**

Participants with T1D had significantly higher glomerular basement membrane (GBM) thickness compared with HCs. T1D participants exhibited lower insulin sensitivity and cortical oxidative metabolism, correlating with higher insulin sensitivity. Proximal tubular transcripts of TCA cycle and oxidative phosphorylation enzymes were lower in T1D. Spatial metabolomics showed reductions in tubular TCA cycle intermediates, indicating mitochondrial dysfunction. The Slingshot algorithm identified a lineage of proximal tubular cells progressing from stable to adaptive/maladaptive subtypes, using pseudotime trajectory analysis, which computationally orders cells along a continuum of states. This analysis revealed distinct distribution patterns between T1D and HCs, with attenuated oxidative metabolism in T1D attributed to a greater proportion of adaptive/maladaptive subtypes with low expression of TCA cycle and oxidative phosphorylation transcripts. Pseudotime progression associated with higher HbA1c, BMI, and GBM, and lower insulin sensitivity and cortical oxidative metabolism.

**CONCLUSION:**

These early structural and metabolic changes in T1D kidneys may precede clinical DKD.

**TRIAL REGISTRATION:**

ClinicalTrials.gov NCT04074668.

**FUNDING:**

University of Michigan O’Brien Kidney Translational Core Center grant (P30 DK081943); CROCODILE studies by National Institute of Diabetes and Digestive and Kidney Diseases (NIDDK) (P30 DK116073), Juvenile Diabetes Research Foundation (JDRF) (2-SRA-2019-845-S-B), Boettcher Foundation, Intramural Research Program at NIDDK and Centers for Disease Control and Prevention (CKD Initiative) under Inter-Agency Agreement #21FED2100157DPG.

## Abstract 2

Young adults with type 1 diabetes showed reduced kidney oxidative metabolism linked to insulin resistance, potentially contributing to early diabetic kidney injury.

## Introduction

Insulin resistance (IR) is prevalent in children and adults with type 1 diabetes (T1D) and often accompanies intensive glycemic therapy. The presence of IR may promote oxidative stress and inflammation, potentially contributing to mitochondrial dysfunction (1–4). Diabetic kidney disease (DKD) is common in people with longstanding T1D, often progresses to kidney failure requiring dialysis or kidney transplantation, and markedly augments risks of cardiovascular disease and premature death (5, 6). IR is implicated in the pathogenesis of DKD, yet the molecular and metabolic underpinnings of IR on kidney function and structure are incompletely understood. Characterizing the molecular and metabolic effects of IR on early kidney injury within the context of contemporary diabetes management (e.g., continuous glucose monitoring [CGM] and automated insulin delivery systems) and identifying modifiable risk factors could lead to more effective and targeted therapies to prevent development of progressive DKD.

This study sought to comprehensively characterize whole-body insulin sensitivity and kidney oxidative metabolism and how they relate to kidney structure, body habitus, and glycemic control in young adults with and without T1D and preserved kidney function. We enrolled young adults with T1D prescribed contemporary diabetes technology who had normotension and normal kidney function without albuminuria. This approach allowed us to study early metabolic perturbations prior to the onset of clinically apparent kidney disease while minimizing the confounding effects of important comorbidities on these perturbations. In 30 young adults with T1D and 20 healthy controls (HCs), we implemented rigorous metabolic and kidney phenotyping including measuring whole-body insulin sensitivity by hyperinsulinemic-euglycemic clamps, glomerular filtration rate (GFR) by iohexol clearance, renal plasma flow (RPF) by *p*-aminohippurate clearance, and kidney perfusion and oxygenation by multiparametric kidney MRI. A subset of participants also underwent optional research kidney biopsies (*n* = 40) with state-of-the-art molecular (single-cell RNA-Seq [scRNA-Seq]), metabolic (spatial metabolomics), and morphometric interrogation of kidney tissue as well as quantification of kidney oxidative metabolism using PET (*n* = 23) to determine ^11^C-acetate turnover in the TCA cycle. Additionally, we performed pseudotime trajectory analysis using the Slingshot algorithm to identify distinct patterns of cellular progression and their association with metabolic and structural parameters.

## Results

### Clinical characteristics of cohort.

Participant characteristics are summarized in Table 1. Participants with T1D were similar to the HCs in age (24 ± 3 vs. 25 ± 3 years) and BMI (25 ± 3 vs. 23 ± 2 kg/m^2^) with an even distribution of men and women in each group. Participants with T1D had a diabetes duration of 13 ± 5 years, an average HbA1c of 7.5 ± 1.0, compared with 5.1 ± 0.3% in HCs, and 97% were using traditional insulin pumps or automated insulin delivery systems at time of study with continuous glucose monitor glucose (CGMg). By design, none of the participants exhibited evidence of elevated albuminuria or impaired estimated GFR (e.g., <60 mL/min per 1.73 m^2^) at screening. Urine albumin excretion rates at 24-hour collection were similar between participants with and without T1D. Total cholesterol, LDL cholesterol (LDL-C), and triglycerides were similar between participants with and without T1D. HDL cholesterol (HDL-C) was slightly higher in participants with T1D (54.5 ± 12.7 vs. 47.5 ± 9.9 mg/dL, *P* = 0.05). None of the participants were treated by renin-angiotensin-system–acting (RAS-acting) agents (angiotensin-converting enzyme inhibitors [ACEi] or angiotensin II receptor blockers [ARB]), statins, metformin, sodium-glucose cotransporter-2 inhibitors (SGLT2i), or glucagon-like peptide-1 receptor agonists (GLP1-RA).

### Body composition, whole-body, and adipose insulin sensitivity.

Participants with T1D demonstrated greater body fat percentages (30.4% ± 7.5% vs. 25.0% ± 6.1%, *P* = 0.01) and fat mass (22.8 ± 6.9 vs. 16.2 ± 4.3 kg, *P* < 0.0001) on dual energy x-ray absorptiometry (DXA). No significant differences in lean mass and muscle mass were observed between the 2 groups (Table 1). Participants with T1D were less insulin sensitive than the HC group (7.8 ± 2.6 vs. 14.3 ± 4.0 mg/kg/min, *P* < 0.0001) during hyperinsulinemic-euglycemic clamps, and the difference between the 2 groups remained similar in magnitude and significance after normalizing by lean mass and adjusting for steady -state insulin concentrations (Table 1). These differences also remained significant after adjusting for body fat percentage.

Baseline nonesterified fatty acid (NEFA) concentrations were higher in those with T1D (1164.9 ± 640.2 vs. 808.6 ± 280.8 μM, *P* = 0.03). Additionally, NEFA suppression in response to 8 mU/m^2^/min insulin infusion was attenuated in T1D versus HCs (LSmean [95% CI]: 55.1% [47.8, 62.4] vs. 80.4% [71.4, 89.3]%, *P* = 0.001) and 40 mU/m^2^/min (LSmean [95% CI]: 89.9% [85.7, 94.1] vs. 96.9% [91.8, 102], *P* = 0.047) after adjusting for baseline NEFA concentrations and body fat percentage (Table 1).

### Measured GFR and RPF, and intraglomerular hemodynamic parameters.

Measured GFR was similar in participants with and without T1D (147 ± 22 vs. 145 ± 29 mL/min, *P* = 0.78) and no participant in either group had a measured GFR less than 90 mL/min. Likewise, RPF was similar in the 2 groups, although renal vascular resistance (RVR) was lower and glomerular pressure (P_GLO_) was higher in T1D compared with HCs (Table 2).

### Kidney structure and function parameters by multiparametric MRI.

Total kidney volume (TKV) and apparent diffusion coefficients (ADC) by MRI were similar between T1D and HCs (Table 2). Participants with T1D exhibited lower kidney perfusion by pseudo-continuous arterial spin labeling (pCASL) MRI than HCs (196 ± 68 vs. 243 ± 48 mL/min/100 g, *P* = 0.01). We found no difference in cortical oxygenation by blood oxygen level–dependent (BOLD) MRI between participants with and without T1D (Table 2). Medullary oxygenation was higher (i.e., lower R2*, hyperoxia) in individuals with T1D than in HCs (*P* = 0.04) and was accentuated when adjusting for kidney perfusion (geometric mean [95% CI] R2*: 26.2 [25.0, 27.4] vs. 28.4 [26.9, 29.8] s^–1^, *P* = 0.03). Furosemide-suppressible oxygen consumption (FSOC) by BOLD MRI was similar between the 2 groups and remained similar after adjusting for kidney perfusion.

### Assessment of kidney oxidative metabolism by ^11^C-acetate PET (substudy).

**Figure 1:**
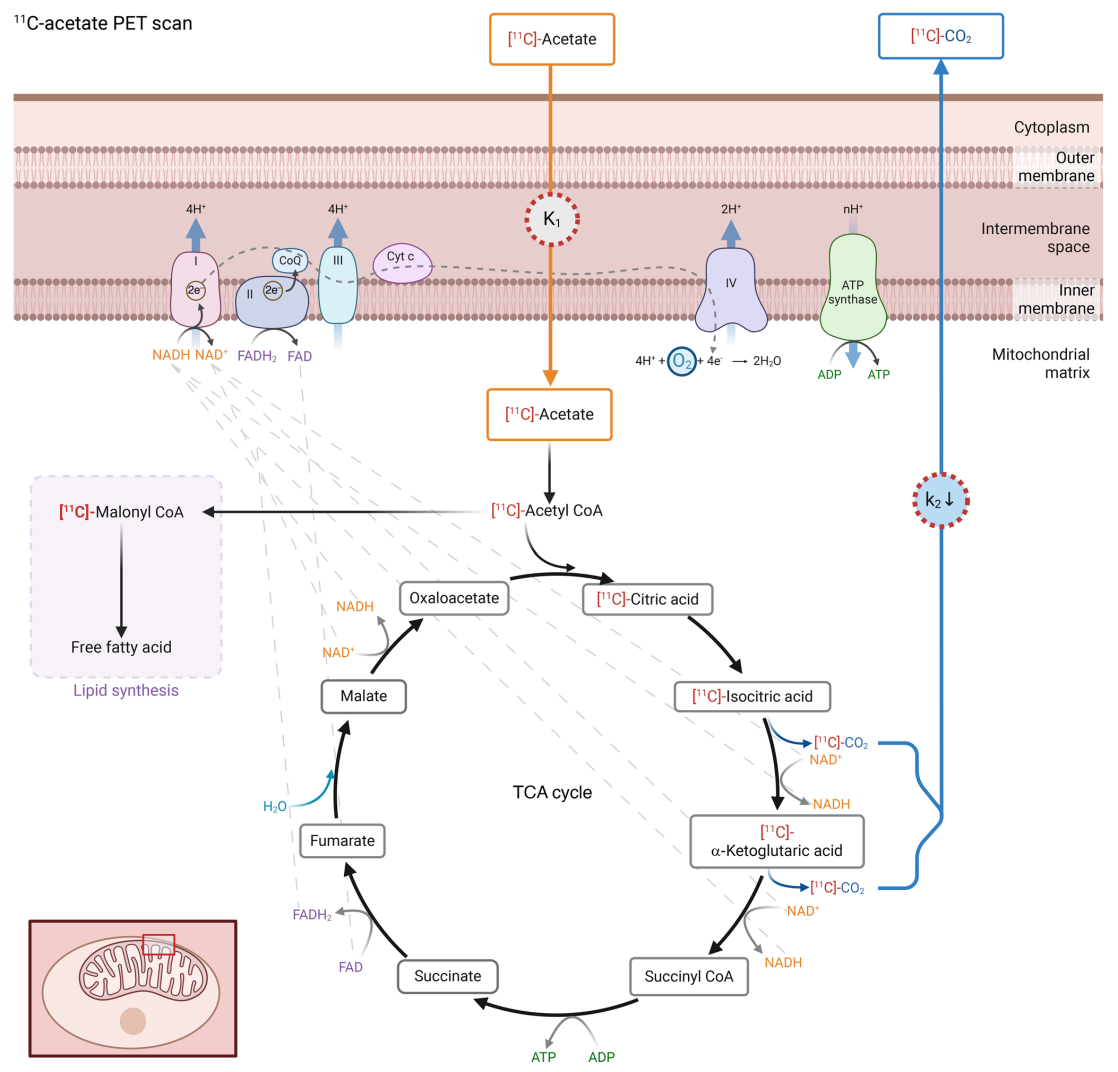
^11^C acetate PET scanning to assess kidney oxidative metabolism. Visual illustration of *K_1_* and *k_2_* in the context of TCA cycle. Refer to Table 2 for results. *t* Tests were performed to compare means of *K_1_* and *k_2_* between HC and T1D. HC and T1D had similar ^11^C-acetate uptake (*K_1_*), but T1D had a lower rate of tracer clearance, estimated by the rate of CO_2_ production (*k_2_*).

Sixteen participants with T1D and 11 HCs underwent the optional ^11^C-acetate PET/CT scan (*n* = 27). Cortical uptake of ^11^C-acetate tracer (*K*_1_) was similar in participants with T1D and HC. In voxel-wise models, average cortical oxidative rate (*k*_2_) was approximately 11% lower in T1D compared with HCs (average: 0.16 ± 0.02 vs. 0.18 ± 0.02 min^–1^, *P* = 0.04, Table 2 and Figure 1). Average medullary oxidative rate (*k*_2_) was approximately 17% lower in T1D compared with HCs in voxel-wise models (average: 0.15 ± 0.03 vs. 0.18 ± 0.02 min^–1^, *P* = 0.04, Table 2 and Figure 1). The difference in cortical and medullary oxidative rates did not reach statistical significance in the regions of interest (ROI) pharmacokinetic (PK) analyses, suggesting regional variations in kidney oxidative metabolism that may not be apparent in global analysis.

Cortical oxidative rate (*k*_2_) correlated with higher whole-body insulin sensitivity (*r*: 0.42, *P* = 0.04), and remained significant after adjusting for age, sex, and HbA1c (β ± SE: 0.005 ± 0.002, *P* = 0.02). Similar associations were observed for medullary *k*_2_ when adjusting for age, sex, and HbA1c.

### Morphometric evaluation of kidney tissue (substudy).

**Figure 2:**
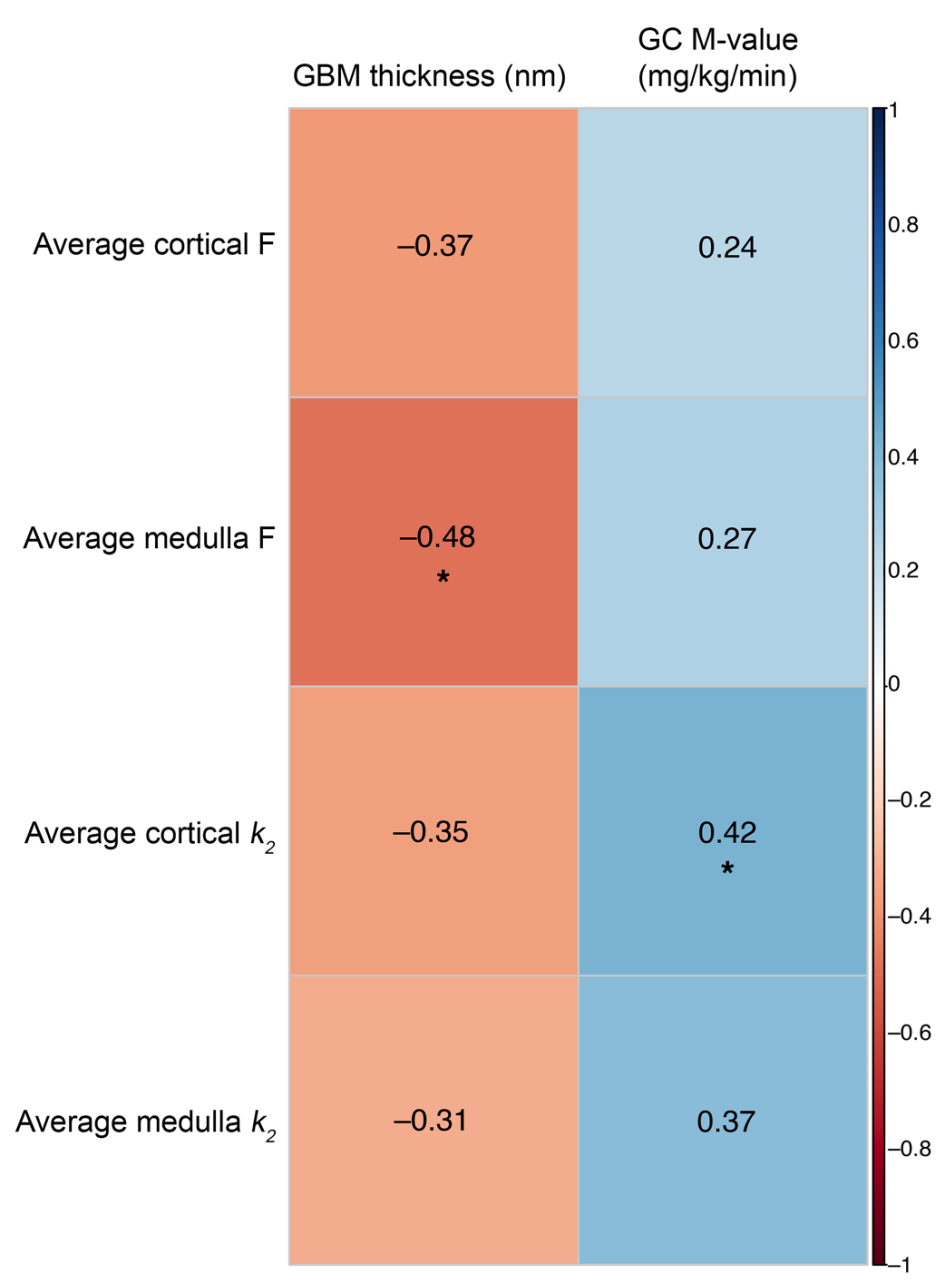
Heatmap of correlations between GBM thickness and M-value with PET parameters. The color gradient in the figure represents the direction of correlation, with negative correlations depicted in red and positive correlations in blue. Spearman’s correlation analysis was performed and the correlation coefficient is presented as numerical text in the boxes. Significant correlation coefficients (*P* < 0.05) are denoted by asterisks. GC, glucose corrected; F, perfusion; *k_2_*, tracer clearance rate.

Twenty-eight participants with T1D and 12 HCs underwent optional research kidney biopsies (*n* = 40). By light microscopy, fractional interstitial area, glomerular tuft area, glomerular volume, and mesangial matrix volume and index were similar between the 2 groups (Table 3). Glomerular nuclear count was lower in participants with T1D compared with HCs. By electron microscopy, glomerular basement membrane (GBM) thickness was significantly higher in T1D than in HCs (median GBM width [p25, p75]: 521.5 [438.1, 646.8] vs. 446.7 [409.1, 473.5], *P* = 0.007). Next, we examined associations between GBM thickness and PET parameters. GBM thickness correlated inversely with kidney medullary perfusion by ^11^C-acetate PET (*r*: –0.48, *P* = 0.03). All correlations are summarized in Figure 2. All correlations adjusted for sex, HbA1c, and age are summarized in Supplemental Figure 1 (supplemental material available online with this article; https://doi.org/10.1172/JCI183984DS1).

### Transcriptomic interrogation of kidney tissue (substudy).

The kidney tissue obtained from the biopsies also underwent transcriptomic and metabolomic interrogation. scRNA-Seq profiles were generated from kidney biopsies from each of the participants (*n* = 40); 85,310 cells passed quality control (QC) requirements and were annotated to 19 clusters, representing all major cell types in the nephron (Supplemental Figures 2 and 3). Each cell cluster had a robust representation in participants with and without T1D (Supplemental Figures 2 and 3).

**Figure 3:**
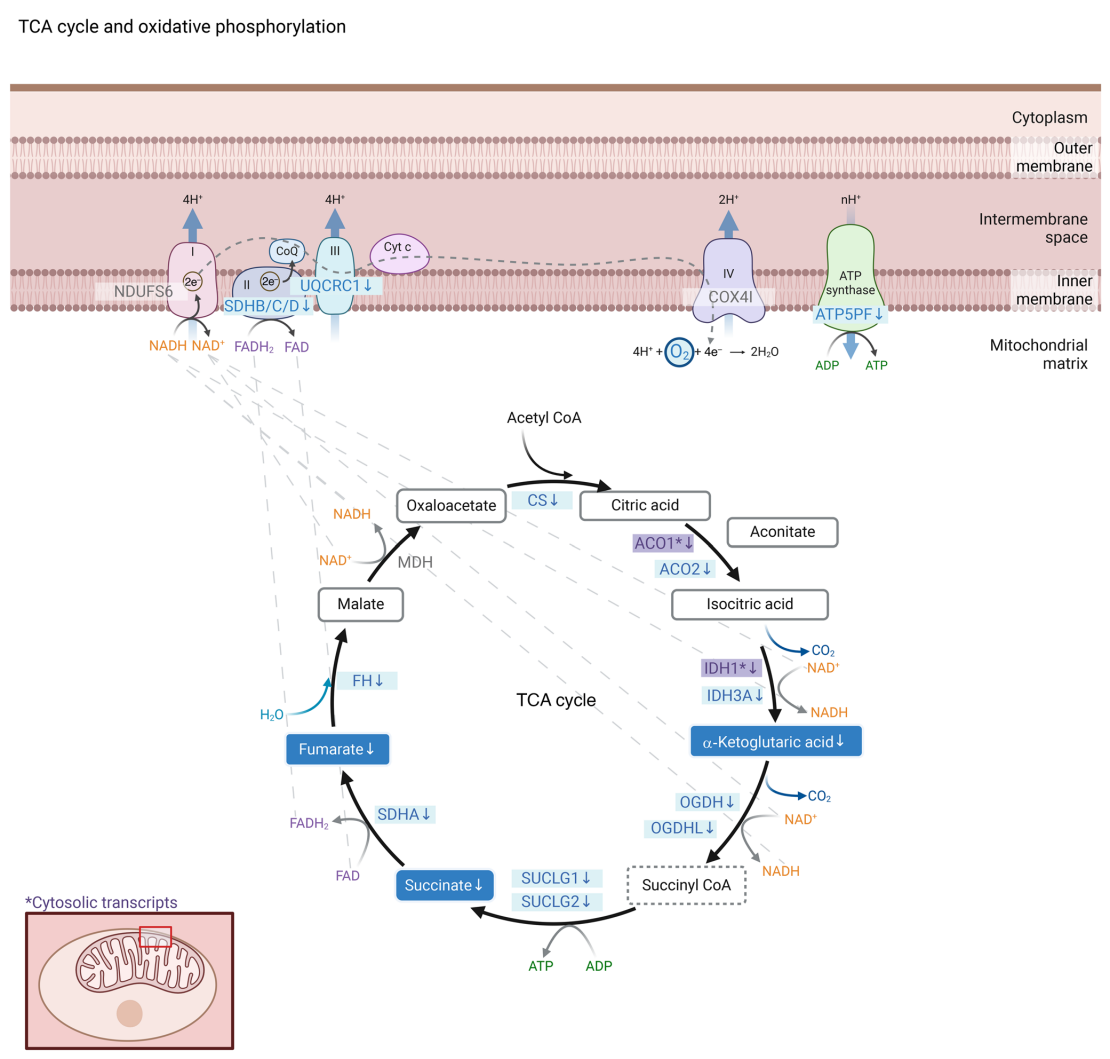
PT transcripts catalyzing the steps of TCA cycle and oxidative phosphorylation and intrarenal TCA metabolites. Visual illustration of PT transcripts and kidney tissue metabolites involved in the TCA cycle and oxidative phosphorylation. Refer to Table 4 and Table 5 for results from analyses. Transcripts and metabolites are shaded in blue (or purple for cytosolic transcripts) when T1D has lower expression/relative abundance relative to HC. Succinyl CoA was not measured in the kidney tissue metabolomics and is shown in a dotted bordered box.

Most proximal tubule (PT) transcripts of the enzymes catalyzing the steps of the TCA cycle, including citrate synthetase (CS), aconitase (*ACO1*, *ACO2*), isocitrate dehydrogenase (*IDH1*, *IDH3A*), oxoglutarate dehydrogenase (*OGDH*, *ODGHL*), succinate-CoA ligase (*SUCGL1*, *SUCGL2*), succinate dehydrogenase complex, subunit A (*SDHA*), and fumarate hydratase (*FH*), were lower in T1D versus HCs (Table 4, Figure 3, and Supplemental Figure 4). Several PT transcripts of oxidative phosphorylation, such as *SDHB*, *SDHC*, *SDHD* (complex II), *UQCRC1* (complex III), and *AT5PF* (complex V), were also lower in T1D versus HCs (Table 4, Figure 3, and Supplemental Figures 4 and 5).

### Metabolomic interrogation of kidney tissue (substudy).

**Figure 4:**
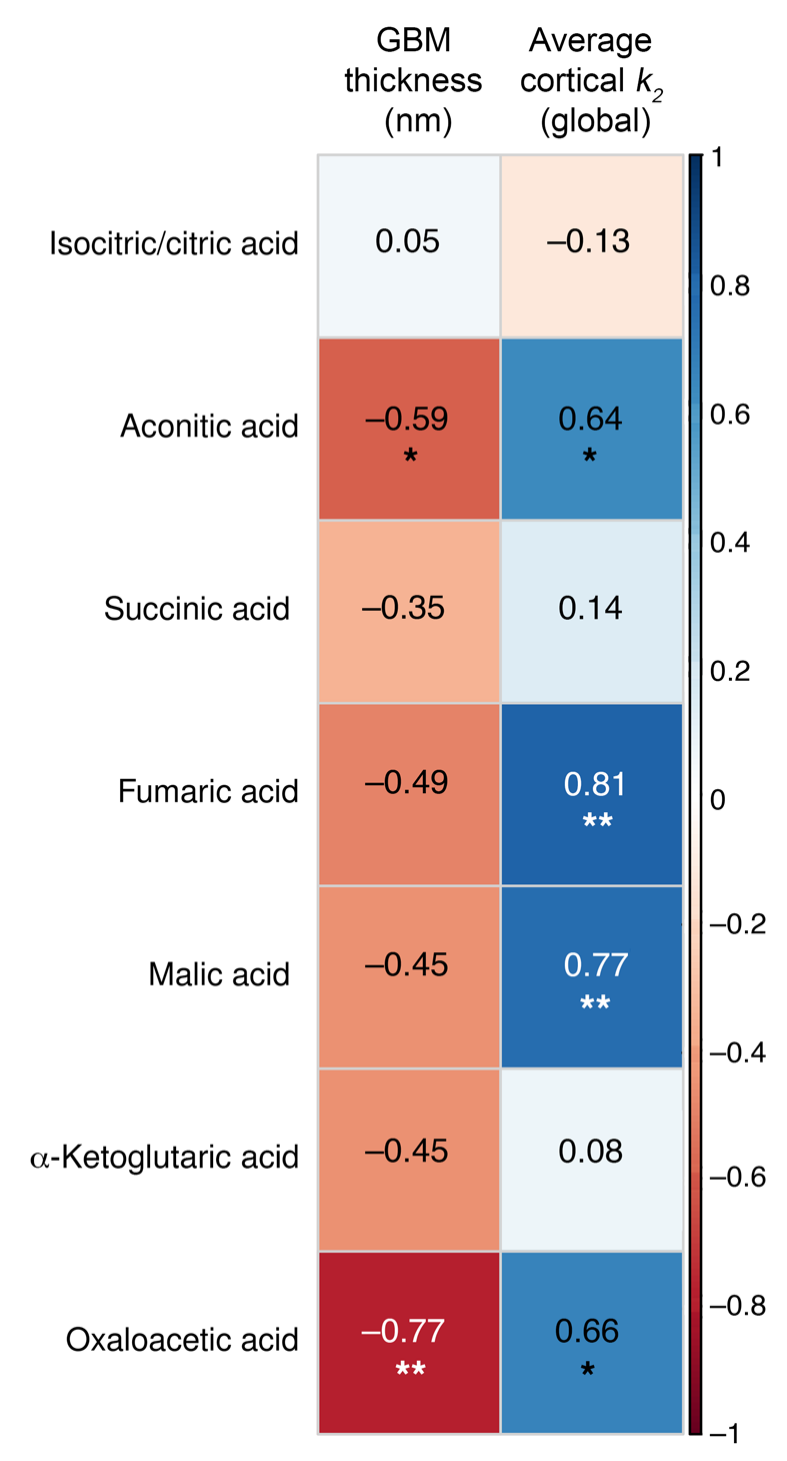
Heatmap of correlations between GBM thickness and cortical *k_2_* with kidney tissue metabolites. The color gradient in the figure represents the direction of correlation, with negative correlations depicted in red and positive correlations in blue. Spearman’s correlation analysis was performed and the correlation coefficient is presented in black text. Significant correlation coefficients (*P* < 0.05) are denoted by asterisks.

Kidney tissue from the subset of participants with spatial metabolite data (T1D, *n* = 16; HC, *n* = 7) revealed significantly lower tubular relative abundance of the TCA metabolites α-ketoglutaric acid (–5.48 [95% CI: –10.2, –0.73; *P* = 0.03]), succinic acid (–8.66 [95% CI: –16.5, –0.80, *P* = 0.04]), and fumaric acid (–8.63 [95% CI: –16.5, –0.81; *P* = 0.04]) in T1D compared with HCs (Table 5 and Figure 3). Next, we examined correlations among GBM thickness, cortical oxidative rate (k_2_), and tubular TCA metabolites. GBM thickness correlated inversely with aconitic acid (*r*: –0.59, *P* = 0.03) and oxaloacetic acid (*r*: –0.77, *P* = 0.002). Cortical oxidative rate (k_2_) correlated positively with aconitic acid (*r*: 0.64, *P* = 0.048), fumaric acid (*r*: 0.81, *P* = 0.005), malic acid (*r*: 0.77, *P* = 0.009), and oxaloacetic acid (*r*: 0.66, *P* = 0.04). All correlations are summarized in Figure 4.

Spatial metabolic analysis also revealed lower TCA cycle intermediates in regions of tubular pathology in participants with T1D compared with HCs (Supplemental Figure 6). Representative periodic acid–Schiff (PAS) images showed atrophic tubules in T1D and normal tubules in HCs (Supplemental Figure 7). The semiquantitative analysis showed that the percentage of atrophic tubules was significantly higher in T1D (12.4%) compared with HCs (0.5%, *P* = 0.0028; Supplemental Figure 7).

### Trajectory analysis (substudy).

**Figure 5:**
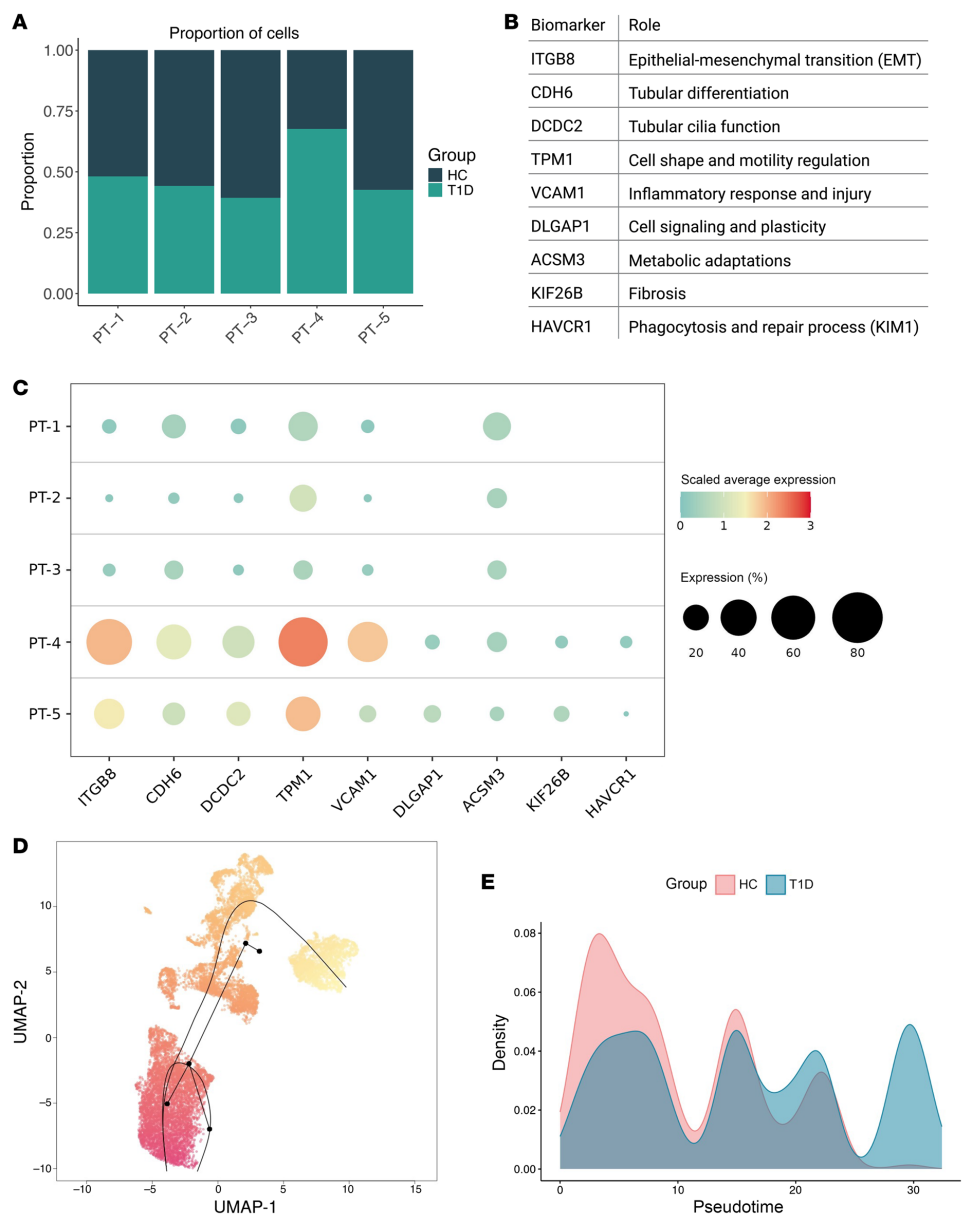
Proportion and expression of proximal tubular cell subtypes with adaptive/maladaptive markers and trajectory analysis. (**A**) Proportion of cells from HC and T1D in each PT cell subtype is shown. The highest proportion of T1D cells was in the PT-4 subtype, and the lowest proportion of T1D cells was in the PT-3 subtype. (**B**) Definition of each adaptive/maladaptive biomarkers as shown in **C**. (**C**) Average expression of adaptive/maladaptive markers (*ITGB8*, *CDH6*, *DCDC2*, *TPM1*, *VCAM1*, *DLGAP1*, *ACSM3*, *KIF26B*, and *HAVCR1*) were higher in PT-4 and PT-5 subtypes compared with PT1-3 subtypes. Higher expression is presented by bigger circles and color closer to red. (**D**) UMAP of PT cells with 2 lineages identified by Slingshot. The first lineage inferred trajectory progressed from PT-3, -2, -5, and -4 (longer lineage), and the other from PT-3, -2, and -1. The first lineage was the main focus for this study. (**E**) Density plot of cell expression across pseudotime revealing differential progression between T1D and HC. PT, proximal tubular.

The scRNA-Seq analysis of PT cells identified 5 distinct subtypes (PT1–5). The proportion of cells from individuals with T1D was highest in the PT-4 subtype and lowest in the PT-3 subtype compared with HCs (Figure 5A and Supplemental Figure 8). The PT-4 and PT-5 subtypes exhibited higher average expression of adaptive/maladaptive markers, including ITGB8, CDH6, DCDC2, TPM1, VCAM1, DLGAP1, ACSM3, KIF26B, and HAVCR1, compared with the PT1–3 subtypes (Figure 5, B and C) (7). Trajectory analysis using the Slingshot algorithm identified 2 lineages, with the main lineage progressing from PT-3, -2, -5, to -4 (Figure 5E). Density plots of cell expression across pseudotime revealed differential progression between T1D and HCs along this lineage as determined by the asymptotic 2-sample Kolmogorov-Smirnov test (*P* < 0.001; Figure 5E). Examination of transcripts involved in the TCA cycle revealed differential expression between HC and T1D groups within each PT subtype (Supplemental Figures 9 and 10). The PT-4 subtype, which had the highest proportion of cells from T1D, showed lower expression of several TCA cycle transcripts in T1D compared with HCs.

**Figure 6:**
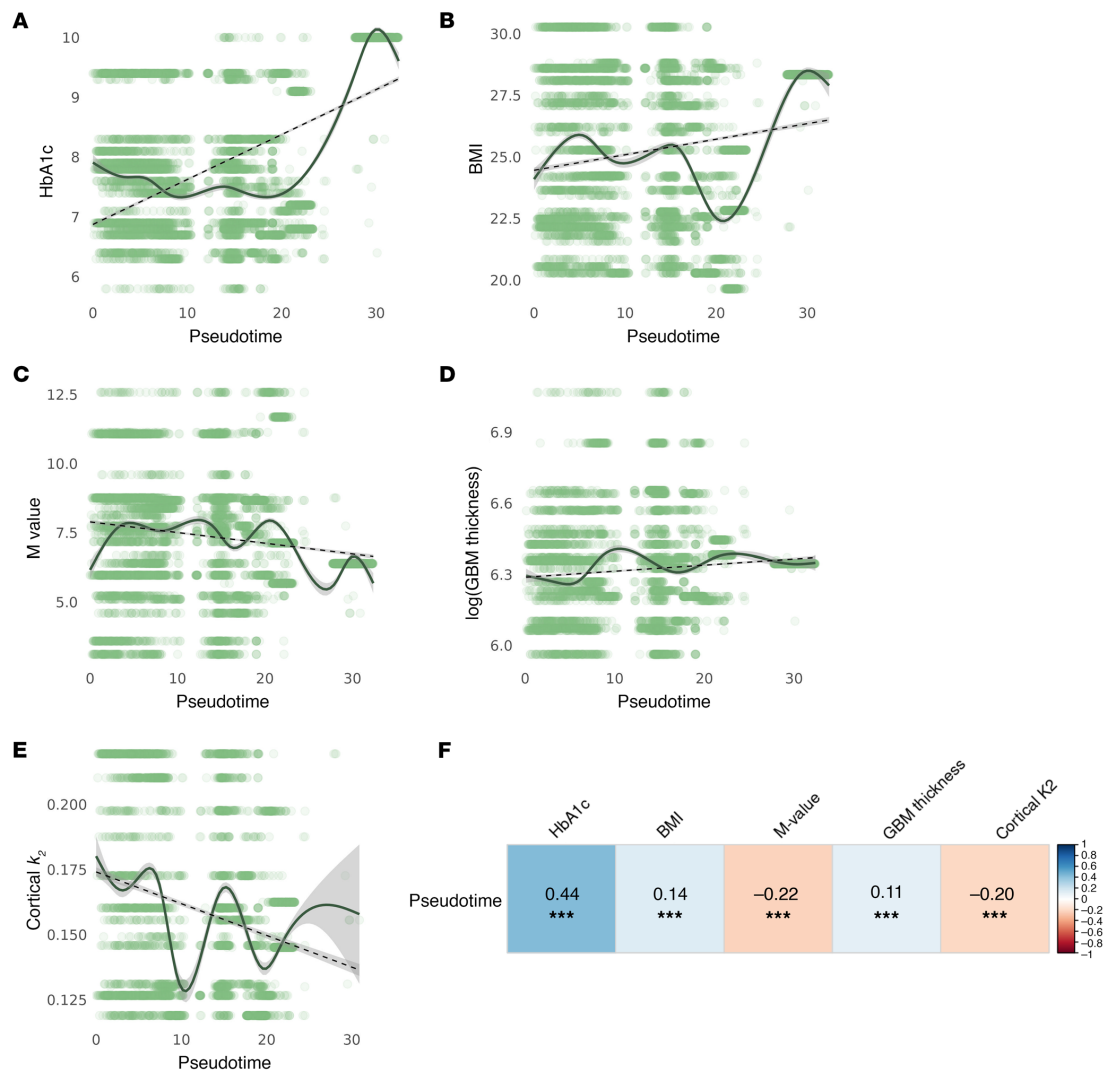
Clinical measures along pseudotime. (**A**–**E**) HbA1c, BMI, M-value, log (GBM thickness), and average cortical *k_2_* are plotted along the trajectory of pseudotime. (**F**) Spearman’s correlations were calculated for each clinical measure with pseudotime, revealing positive correlations with HbA1c, BMI, and GBM thickness and negative correlations with M-value and cortical *k_2_*. HbA1c, glycated hemoglobin; *k_2_*, cortical oxidative metabolism.

**Figure 7:**
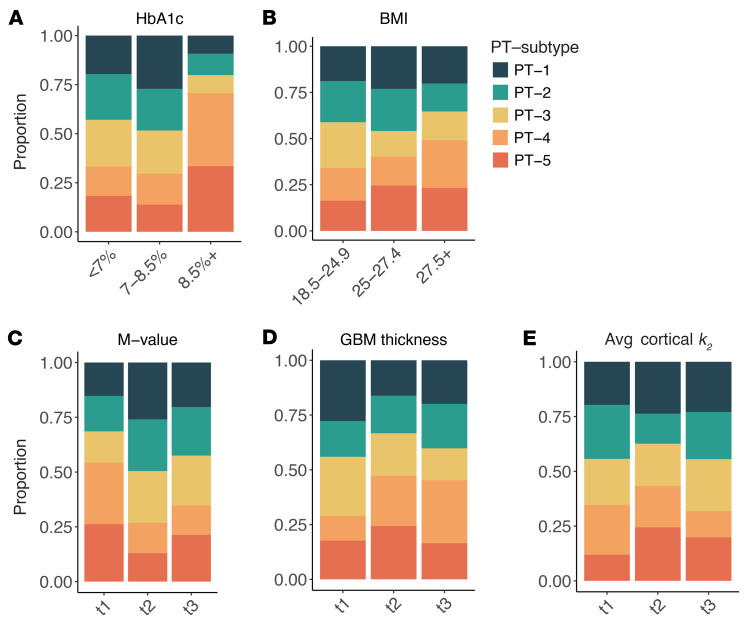
Proportion of PT cell subtypes in categories of clinical measures. Proportion of PT-4 cells is higher for higher categories of HbA1c (**A**), BMI (kg/m^2^, **B**), and GBM thickness (**D**), and lower for higher categories of M-value (**C**) and average cortical *k_2_* (**E**). HbA1c cutoffs were based on the American Diabetes Association guidelines. Tertiles were used for M-values (t1: [3.1, 7.7%]; t2: (7.7, 11.3%]; t3: (11.3, 25.5%]), GBM thickness (t1: [300.0, 488.5 nm]; (488.5, 582.2nm]; (582.2, 1026 nm]), and average cortical *k_2_* (t1: [0.119, 0.153]; t2: (0.153, 0.187]; t3: (0.187, 0.219] min^–1^). *k_2_*, cortical oxidative metabolism.

Analysis of clinical measures along the pseudotime trajectory revealed positive correlations between pseudotime and HbA1c, BMI, and GBM thickness as well as negative correlations with whole-body insulin sensitivity (M-value) and cortical oxidative rate (*k*_2_) (Figure 6). The proportion of PT-4 cells was higher in categories with higher HbA1c, BMI, and GBM thickness and lower in categories with higher M-value and average cortical *k*_2_ (Figure 7).

## Discussion

Our comprehensive analysis reveals insight into the diverse effects of T1D on kidney oxidative metabolism. The most salient findings include impaired kidney oxidative metabolism in young adults with T1D by PET imaging, which is associated with whole-body IR. The in-depth analysis of the kidney tissue transcriptome and metabolome uncovered perturbations in both TCA cycle and oxidative phosphorylation-related transcripts and TCA cycle metabolites, findings that are congruous with the data acquired from the functional PET examinations. The trajectory analysis using the Slingshot algorithm identified a lineage of PT cells progressing from stable to adaptive/maladaptive subtypes, with T1D cells exhibiting a more advanced pseudotime distribution along this lineage compared with HCs. These findings suggest that the progression toward adaptive/maladaptive proximal tubular subtypes, characterized by higher expression of injury markers and lower expression of TCA cycle transcripts, may contribute to the development of early diabetic kidney injury in T1D. These findings help us understand early metabolic changes that occur in the kidneys in people living with T1D and which may increase the risk of DKD.

DKD is a common and serious complication in people with T1D. Apart from the risk of developing kidney failure, it markedly augments risks of cardiovascular disease and premature death (5, 6). Kidney disease typically develops at between 5 and 30 years of T1D duration depending upon the extent to which glycemic control is achieved and maintained (8, 9). Recent advances in CGM and automated insulin delivery systems facilitate attainment of strict glycemic control. In response to these improvements, clinical manifestations of DKD appear to be evolving. The incidence of DKD is declining, though it remains high (8–12). In addition, reduction of GFR now occurs frequently in the absence of heavy proteinuria, and rates of kidney failure have decreased over time in some populations (13–15). However, the pathologic and metabolic causes of these changes are unknown. Intensive glycemic control remains a key target to prevent DKD in T1D, alongside other important interventions such as RAS blockade (16, 17). Despite modern advances in glycemic monitoring and therapy, euglycemia remains elusive, substantial residual risk remains, and complementary therapies are needed. Furthermore, intensive glycemic control in the Diabetes Control and Complications Trial (DCCT) was associated with weight gain in a subset of participants, accompanied by worsening IR, central obesity, lipids, blood pressure, inflammation, and albuminuria, all of which may undermine positive effects of improved glycemic control (1–4).

IR is associated with increased kidney tubular glucose and NEFA uptake while impairing glucose and NEFA oxidation, TCA cycle turnover, and oxidative phosphorylation (18–28). These metabolic perturbations may predispose to oxidative stress in T1D by magnifying the kidney’s already high energy expenditure while impairing substrate metabolism (29–31). The resultant intracellular accumulation of lipid and glucose intermediates shifts transcription toward suppression of adenosine monophosphate kinase (*AMPK*) and activation of mechanistic target of rapamycin complex 1 activity (*mTORC1*), potentially leading to long-term impairment of TCA cycle turnover and oxidative phosphorylation, and ultimately kidney injury (22–25, 32–38). Our data provide insights into kidney metabolic changes in T1D, aligning with the growing body of research suggesting that perturbed energetics are central mechanisms in the pathogenesis of DKD. We observed decreased cortical and medullary oxidative rates via ^11^C-acetate PET/CT scans, lower PT transcripts of enzymes catalyzing the steps of the TCA cycle and oxidative phosphorylation, and lower tubular TCA intermediates by spatial metabolomics in T1D participants. These findings are consistent with gene expression analyses from kidney biopsies in patients with advanced stages of chronic kidney disease (CKD) without diabetes, which exhibit lower transcripts of TCA cycle enzymes than healthy reference tissue (39), and with lower plasma TCA metabolites documented in young adults with T1D compared with HCs (40). Moreover, the trajectory analysis revealed that the progression toward adaptive/maladaptive proximal tubular subtypes was associated with higher HbA1c, BMI, and GBM thickness as well as lower insulin sensitivity and cortical oxidative metabolism. These results suggest that the metabolic and structural changes observed in T1D may drive the progression of PT cells toward maladaptive states, potentially contributing to the development of DKD. It is crucial to establish these relationships, given that emerging adjunctive therapies proposed to improve insulin sensitivity and kidney metabolism, such as GLP-1RA and SGLT2i, which were previously shown to be strongly kidney protective in type 2 diabetes (T2D), are currently being tested in individuals with T1D (41–43).

Prior gene expression studies have revealed reduction of transcripts catalyzing the proximal half of the TCA cycle as well as reduced corresponding urinary metabolites in advanced DKD in T2D and nondiabetic CKD and murine models (39, 44–46). In our study, we documented these abnormalities in individuals with T1D and preserved kidney function using scRNA-Seq and spatial metabolomics, and demonstrated corresponding functional changes using ^11^C-acetate PET imaging. Importantly, despite participants having normal GFR and no albuminuria, they displayed intraglomerular hypertension and increased GBM thickness, potentially as a compensatory mechanisms against heightened pressure. These observations indicate the presence of early subclinical kidney injury (47). In addition, the observed lower kidney perfusion in participants with T1D, despite similar GFR and RPF levels, may be attributed to microvascular dysfunction and structural changes such as increased tissue resistance, interstitial fibrosis, and GBM thickening (48, 49). These factors can lead to a discrepancy between perfusion and blood flow, highlighting the complex interplay between renal hemodynamics and microvascular health in T1D. We observed differences in oxidative metabolism between cortical and medullary regions consistent with known renal physiology (50–52). MRI-derived R2* values were higher in the medulla, suggesting lower oxygen availability compared with the cortex. PET-derived *k_2_* values showed subtle regional differences, with lower *K_1_* and F values in the medulla indicating reduced perfusion. However, our transcriptomic and metabolomic analyses were limited to cortical tissue, precluding a comprehensive comparison of regional metabolic profiles.

In a prior study examining molecular patterns in kidney tissue from adolescents with T2D we found upregulation of most metabolic transcripts in the PT, including the TCA cycle. The differences observed between T1D in the current study and our previous findings in T2D may be ascribed to variations in participant characteristics, such as age, diabetes duration (2.3 ± 1.8 years in T2D vs. 13.3 ± 5.3 years in T1D), and the presence of severe glomerular hyperfiltration in the T2D cohort (53). Additionally, the differences in pseudotime trajectories between T1D and T2D, specifically the proportion of adaptive/maladaptive proximal tubular cells, which exhibit lower expression of TCA cycle and oxidative phosphorylation genes, may contribute to the discrepancies in gene expression patterns.

This study represents an application of ^11^C-acetate to assess kidney oxidative metabolism in young adults with T1D alongside HCs. The voxel-wise PK model allowed us to account for regional differences in kidney oxidative metabolism. Our PET analyses also provided ^11^C-acetate uptake (*K*_1_) and estimates of kidney perfusion (F). The multiparametric MRI analyses provided comprehensive quantification of kidney volume, tissue characteristics, perfusion, and oxygen availability. We employed gold-standard hyperinsulinemic-euglycemic clamps to assess insulin sensitivity, as well as DXA to quantify lean, fat, and visceral mass. Another notable strength of our study lies in the integration of functional PET data with single-cell transcriptomics and spatial metabolomics. The trajectory analysis using the Slingshot algorithm allowed us to identify distinct patterns of cellular progression and their association with metabolic and structural parameters, providing insights into the potential mechanisms underlying the development of early diabetic kidney injury in T1D. This multimodal approach allowed for a detailed and comprehensive interrogation of kidney oxidative metabolism. While our participants were recruited from a tertiary care center, their characteristics reflect contemporary T1D management standards in the United States. The absence of hyperfiltration aligns with recent T1D studies, suggesting our findings are relevant to understanding kidney health in the modern era of diabetes care (54). Limitations of the study included the small sample size and the measurement of key variable in different subsets of individuals. We did not measure glucagon levels during the clamp studies, which could provide additional insights into glucose homeostasis in participants with T1D. The cross-sectional design of our study, while revealing changes in proximal tubular subtypes suggestive of early injury, precludes establishing a direct link between these early metabolic perturbations and the progression of DKD; longitudinal studies are needed to definitively connect these alterations to disease outcomes. Nonetheless, insights from existing literature highlight the substantial influence of early metabolic disturbances and structural lesions on the development of DKD (55, 56). The transition from metabolic disruptions to detectable histological changes underlines a pathophysiological continuum (56), reinforcing the premise that early identification and intervention targeting these metabolic and histological changes could substantially alter DKD trajectory.

In conclusion, this study offers a comprehensive overview of the early kidney metabolic changes associated with T1D in the modern era of diabetes management. The observed alterations in body composition, insulin sensitivity, and kidney structure, along with the significant differences in kidney oxidative metabolism, provide an expanded perspective on the functional kidney manifestations of T1D. Deep clinical phenotyping and molecular interrogation of the kidney tissue of individuals with T1D demonstrates regional metabolic perturbations before the onset of clinically apparent kidney disease. The trajectory analysis identified a progression of PT cells from stable to adaptive/maladaptive subtypes, with T1D cells exhibiting a more advanced distribution along this lineage. These findings suggest that the metabolic and structural changes observed in T1D may drive the progression of PT cells toward maladaptive states, potentially contributing to the development of DKD. Our results underscore the importance of further targeted research to explore these findings more deeply. While our cross-sectional study provides valuable insights into potential early kidney changes in T1D, we initiated the longitudinal study Pathogenesis of Kidney Disease in Type 1 Diabetes: a Modern Kidney Biopsy Cohort (The PANDA Study) (NCT05319990) to track kidney function, structure, and metabolism over time in a subset of participants from the current study with the goal of establishing the temporal relationships linked to progressive DKD. Additionally, it aims to evaluate the impact of promising T1D therapies, such as GLP-1 receptor agonists (GLP-1RA) and SGLT2i. These efforts will help validate our findings as early predictors of DKD and clarify the sequence of changes leading to clinically apparent disease. This research marks a critical step toward delineating early metabolic risk factors for DKD and refining therapeutic strategies to mitigate kidney injury in this population, ultimately aiming to improve patient outcomes.

## Methods

### Sex as a biological variable.

Male and female participants were enrolled and included in the analysis. Adjusted analyses included sex as a covariate.

### Study design and participants.

**Figure 8:**
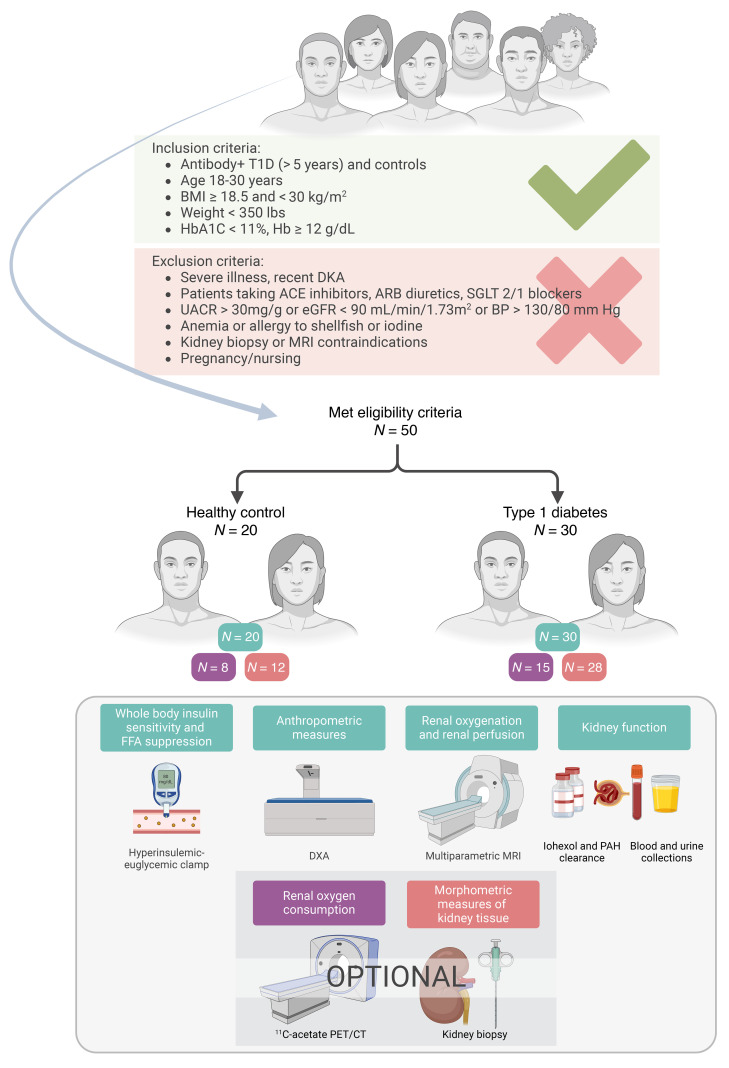
Study design and consort diagram.

Young adults with and without T1D from the Control of Renal Oxygen Consumption, Mitochondrial Dysfunction, and Insulin Resistance (CROCODILE) study (NCT04074668) were included in this analysis. The participants with T1D were recruited from the Barbara Davis Center for Diabetes and the HCs from advertisements. T1D was defined by American Diabetes Association criteria plus the presence of pancreatic autoantibodies, such as glutamic acid decarboxylase, islet cell, zinc transporter 8, and/or insulin autoantibodies. Exclusion criteria are detailed in Figure 8. The participants self-reported their demographics from predefined options as shown in Table 1.

Prespecified primary endpoints of the CROCODILE study included renal oxygen consumption by ^11^C-acetate kidney PET/CT scan, renal oxygen consumption, oxygenation, and perfusion by BOLD and arterial spin labeling (ASL) MRI, and insulin sensitivity by hyperinsulinemic-euglycemic clamp. Secondary endpoints of the study included GFR and RPF from iohexol and P-aminohippurate (PAH) clearance and biomarkers from kidney biopsies. The presented analysis included primary and secondary endpoints of the registered trial.

### Clinical measurements.

Laboratory assays were performed by the University of Colorado Clinical and Translational Research Centers (CTRC) Core Labs. Other fasting laboratory evaluations included total cholesterol, calculated LDL-C, HDL-C, triglycerides, glucose, and HbA1c (DCCT-calibrated); assays were performed by standard methods in the CTRC laboratory. Urine albumin-to-creatinine ratio (UACR) was also determined from a 24-hour urine collection. Participants were provided with urine jugs and were instructed to collect their urine for 24 hours prior to their study visit. The study team documented the start/stop time, date, and total volume of urine. Urine albumin and creatinine were analyzed from a sample of the total collection.

### Hyperinsulinemic-euglycemic clamps and DXA.

A hyperinsulinemic-euglycemic clamp was performed to determine insulin sensitivity with 8 mU/m^2^/min (stage 1) and 40 mU/m^2^/min (stage 2) insulin stages, along with an intravenous infusion of 20% dextrose titrated to maintain euglycemia, based on bedside glucose readings every 5 minutes as in our previous studies (57, 58). Blood samples for insulin were collected at the beginning and during steady state of each stage of the clamp. Steady-state glucose infusion rate (GIR) (mg/lean kg/min) in response to the insulin concentration rate of 40 mU/m^2^/min was used to determine whole-body insulin. Participants also underwent a DXA scan by standard methods on a Hologic device to determine lean and fat mass, as well as regional body composition including visceral fat (59). Muscle mass was calculated by multiplying appendicular lean mass (sum of arm and leg lean mass) and subtracting 0.63 (60).

### GFR and RPF measurements.

Iohexol (Omnipaque 300, GE Healthcare) was administered as a bolus (36 mg/kg) followed by continuous infusion (15 mg/min). PAH (Basic Pharma) was administered as a bolus (11.2 mg/kg) followed by continuous infusion (12.8 mg/min). GFR and RPF were calculated for each urine collection period (U_x_) as follows: flow = volume of urine collection U_x_ (mL)/collection time for U_x_ (min). Uncorrected GFR (or RPF) = concentrations of iohexol (or PAH) in U_x_ (μg/mL) × flow (mL/min)/(P1 + P2)/2 (μg/mL), where P1 and P2 were the plasma concentration of iohexol (or PAH) at the beginning and end of the corresponding timed urine collection (U_x_). The extraction ratio of PAH was adjusted according to GFR (61–63). Iohexol and PAH measurements were performed by high-performance liquid chromatography (HPLC) (Waters) at the NIDDK laboratory in Phoenix, Arizona, as described previously (64–68). Using measurements of GFR, RPF, hematocrit, and total protein, we calculated afferent and efferent arteriolar resistance and intraglomerular pressure by Gomez equations (69–71). We calculated RVR as mean arterial pressure/renal blood flow.

### Multiparametric kidney MRI.

All participants underwent a multiparametric kidney MRI scan on a Siemens Magnetom Skyra 3T scanner at our research imaging center. The scanning protocol included BOLD MRI to estimate fractional oxygen availability (apparent relaxation rate R2* [s^–1^]) (72). A multiple gradient-recalled-echo (mGRE) sequence was used to acquire BOLD images in the coronal plane during breath-hold at end expiration, before and after giving the diuretic furosemide (20 mg i.v. injection). The body coil was used as the transmitter, and the combination of spine and body array coils was used as the receiver. Participants were placed feet first in supine position. The higher the local deoxyhemoglobin in the blood, the higher the R2*, and the lower the local tissue oxygen content. The change in fractional kidney oxygen availability (R2*) in response to furosemide provided an estimate of the oxygen-dependent tubular transport of sodium and kidney oxygen consumption, entitled FSOC. Participants also underwent pCASL MRI to measure kidney perfusion (mL/min/100 g), and ADC to assess tissue stiffness (diffusion weighted imaging scan b-values of 200, 300, 500, 700, and 1000 sec/mm^2^). A custom image processing toolbox using Python (Python Software Foundation) was used to manually define ROI on ADC maps and BOLD images for each slice on both kidneys from T1 or T2 weighted images (72). TKVs were computed. The images were analyzed at NorthShore, and the reader was blinded to the participants’ clinical data.

### ^11^C-acetate PET.

PET scanning was performed under standardized conditions. A Philips Gemini TF 64 was used for PET/CT imaging. A regional CT scan (40 mA·s) centered at the abdomen was performed for attenuation correction and PET registration. Kidney oxidative metabolism was determined by a 342.2 ± 20.6 MBq bolus of ^11^C-acetate followed by a 30-minute list-mode dynamic PET acquisition. A venous sample was collected 25.7 ± 1.1 minutes after radiotracer injection for blood activity calibration. PET images were reconstructed with a 3D row action maximum likelihood algorithm and the following time frames: 12 × 10 seconds, 8 × 30 seconds, 2 × 2 minutes, and 4 × 5 minutes.

PET image processing was performed in PMOD (version 3.7; PMOD Technologies Ltd.). The right and left kidney cortex ROI were defined semi-automatically using iso-contours on an average PET image (from 40 seconds to 4.5 minutes after injection, Supplemental Figure 11). Manual corrections were applied based on the corresponding CT image to remove large blood vessels and nonkidney tissue. The right and left kidney medulla ROIs were segmented manually on the same PET image. The blood signal was obtained from a ROI over the descending aorta, corrected for the presence of ^11^C-labeled metabolites, and scaled based on the blood sample activity.

The ^11^C-acetate turnover in the TCA cycle (73, 74) was estimated for each ROI using a 1-tissue, 2-compartment, PK model with parameters *K*_1_ (tracer uptake), *k*_2_ (tracer clearance, rate of CO_2_ production), and *v*_b_ (blood volume fraction). Blood flow (F) was estimated using the *K*_1_ values and assuming an extraction fraction of 0.52 (74). A voxel-wise PK model provided parametric maps of *K*_1_ and *k*_2_ by fitting a 1-tissue compartment model using linear ridge regression with spatial constraint (Supplemental Figure 12).

### Ultrasound-guided kidney biopsies and tissue processing.

An ultrasound-guided percutaneous kidney biopsy was performed by 1 of 2 highly experienced interventional radiologists. Per local protocol, up to 4 passages were allowed to obtain 3 biopsy cores. Each core was immediately assessed for the presence of cortex by gross examination and digital imaging. Kidney tissue was placed in specific fixatives and shipped to the University of Michigan, according to a modified version of the Kidney Precisions Medicine Project (KPMP) pathology protocol (https://repository.niddk.nih.gov/media/studies/kpmp/KPMP_Pathology-MOP.pdf).

### Quantitative morphometrics.

Light microscopy sections were assessed for pathologic diagnosis. For quantitative assessment of glomerular and mesangial volume and mesangial nuclear count, all glomeruli present in a 3 μm formalin-fixed paraffin-embedded section of each specimen were stained with periodic acid–Schiff and assessed using quantitative morphometrics as previously described (75, 76). Mesangial index was expressed as percentage of mesangial area per glomerular volume. GBM width was measured on electron microscopic sections.

### Sample processing and scRNA-Seq of kidney tissue.

scRNA-Seq profiles were obtained using KPMP protocols. Briefly, single cells were isolated from frozen tissues using Liberase TL at 37°C for 12 minutes. The single-cell suspension was immediately transferred to the University of Michigan Advanced Genomics Core facility for further processing. Sample demultiplexing, barcode processing, and gene expression quantifications were performed with the 10X Cell Ranger, version 6, pipeline using the hg38 GRCh38-2020-A reference genome (77–79). To remove ambient mRNA from the data, the cell ranger count matrices were processed using SoupX with default parameters. The resulting matrices were processed as previously described (80), whereby cells were included only if gene counts were between 500 and 5,000, with fewer than 50% mitochondrial genes. Individual matrices were then integrated using RunHarmony embedded in Seurat, version 4.0.0. Clusters were annotated based on previously established kidney cell markers (Supplemental Figures 1 and 2) (80, 81). Adaptive/maladaptive states were identified and defined on the basis of previous studies and known features of injury (7).

### Spatial metabolomics of kidney tissue.

Kidney tissue samples were frozen in liquid nitrogen and stored at –80°C until analysis. A multimodal imaging approach was developed to investigate regional localization (e.g., tubular cells) of metabolites in kidney sections (82). Briefly, snap-frozen kidney tissues were cryotome sectioned at 10 μm thickness and thaw mounted onto ITO-coated conductive glass slides. Tissue sections were scanned by bright-field (BF) and autofluorescence (AF) microscopy (Zeiss Axioscan 7; ×20 magnification), which outlined glomeruli and tubules, and then were coated using a robotic sprayer (TM-Sprayer) with 1,5-diaminonaphthalene for negative ion mode analysis. After matrix application, the slide was loaded into the slide holder in the matrix assisted laser desorption/ionization (MALDI) injector (SpectroGlyph LLC) (83). Mass spectrometry imaging (MSI) data were acquired using MALDI Injector Software,version 1.3.1.1537, at 20 μm spatial resolution coupled to a Thermo Scientific Q Exactive HF-X Hybrid Quadrupole-Orbitrap Mass Spectrometer (Thermo Scientific). Targeted metabolite species (e.g., the TCA cycle intermediates and adenine) were investigated in tubular cells of kidneys. Periodic acid–Schiff hematoxylin (PAS-H) staining revealed regions of pathology in serial sections (84). All PAS-H–stained slides were evaluated by an experienced kidney pathologist. Metabolite annotation was performed on METASPACE (85) and further validated by comparing tandem mass spectrometry (MS/MS) spectra with standard compounds or databases. The optical image (AF/BF and PAS-H) was uploaded to METASPACE and SCiLS Lab for visual overlay of metabolites with optical images to provide an assessment of metabolites associated with normal-appearing and pathologic features in tubular cell regions. Detailed procedures for MALDI-MSI are available at https://doi.org/10.17504/protocols.io.bctfiwjn (84).

Quantitative analysis of atrophic tubules in PAS images was also performed. Grid lines were added to PAS images in QuPath to cover whole tissue sections. Five ROIs were randomly selected from each sample (HC, *n* = 3; T1D, *n* = 5) for the counting of numbers of total and atrophic tubules. The percentage of atrophic tubules was calculated based on the ratio between the number of atrophic tubules and the number of total tubules in each ROI. A *t* test was performed for the comparison of percentages of normal and atrophic tubules in HC and T1D.

Missing values of spatial metabolomics of the kidney tissue measurements were imputed using 20% of the minimum measurement of each metabolite under the assumption of missing values caused by low abundance of the metabolites (i.e., below the detection limit). Each tissue sample was measured twice from 2 different sections of 1 kidney biopsy, and data were reported on sections which passed the data QC.

### Power calculation.

Sample size for this study was chosen to provide greater than 80% power to detect a difference of 88 nm or more in GBM width assuming a SD of 78 nm based on prior data (64, 86). In addition, our sample size of 28 adults with T1D and 12 lean controls yields 80% power to detect a fold change of 0.8 in gene expression using FDR correction for the 17 genes of primary interest in the TCA cycle or the 9 genes of interest in oxidative phosphorylation in targeted analyses.

### scRNA analysis.

Seurat version 4.1.0 was used to analyze single cell transcriptomics. Data were normalized, scaled, and clustered using default embedded functionalities in the Seurat R package. The integrated dataset was used to run the uniform manifold approximation and projection (UMAP), which was visually inspected for clustering of cell types and expression of groups across clusters. Further analysis of the transcriptomics data was filtered to the PT cells, known for their high oxidative metabolic activity within the kidney. We also focused our analysis on transcripts involved in the TCA cycle and oxidative metabolism according to the aims of the grant that funded this work, which complemented our functional evaluation of the kidney’s oxidative metabolism via ^11^C-acetate PET. Differential expression of genes was identified between HC and T1D using a Wilcoxon’s rank sum test.

**Figure 9:**
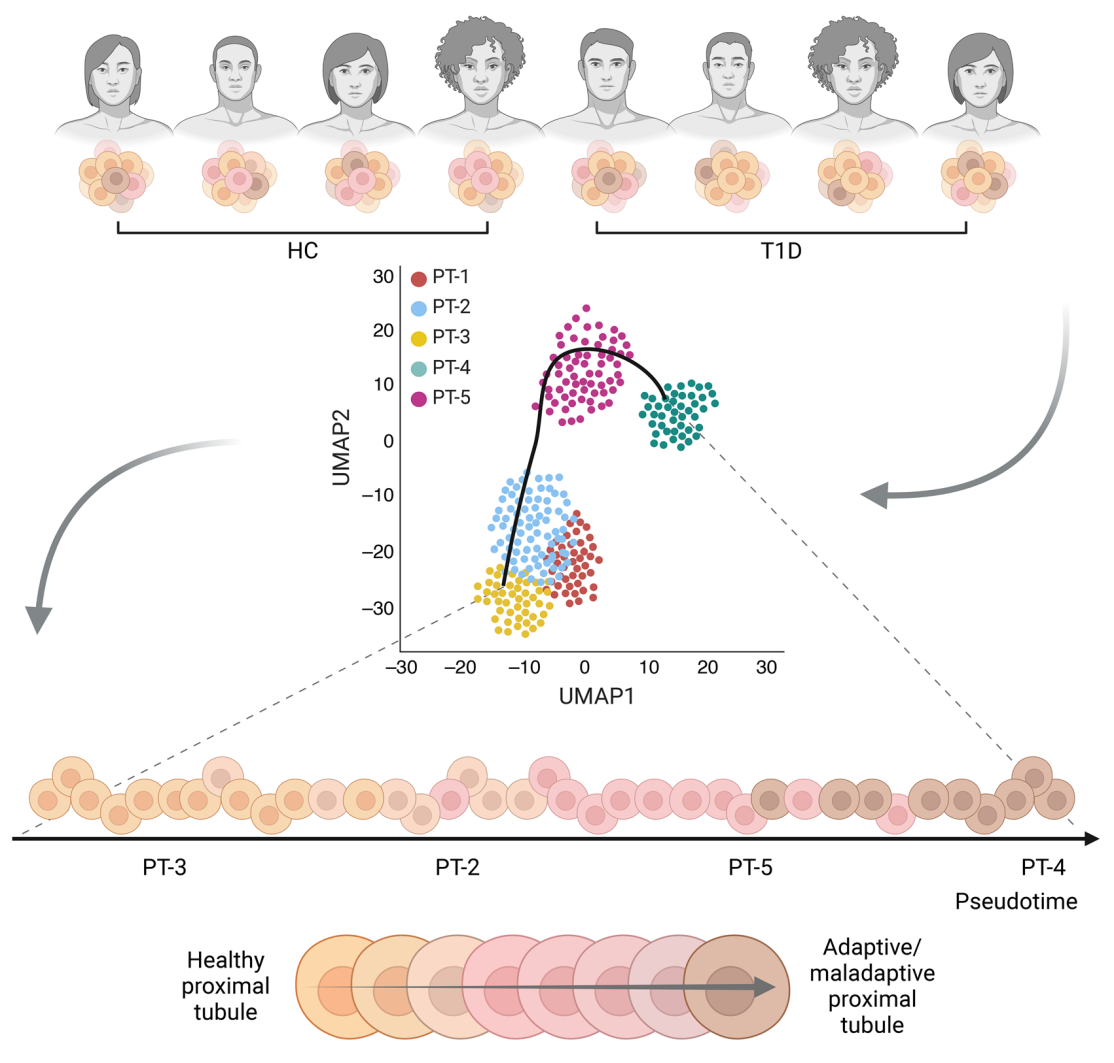
Illustration of the pseudotime analysis concept using Slingshot. Illustration of the pseudotime analysis concept. Cells were collected from independent participants with varying health progression (e.g., diabetes duration and progression). Pseudotime was inferred using the minimal spanning tree method in Slingshot from UMAP. Healthy PTs are indicated in orange, transitioning to adaptive/maladaptive PTs shown in brown.

Following clustering with Seurat, trajectory inference analysis was performed on PT cells only using Slingshot, version 2.12.0. The clusters generated from Seurat were input into the Slingshot function, specifying PT-4 as the end cluster based on its known biological relevance as adaptive/maladaptive cells (Figure 5B). PT-4 cells were assumed to represent the most “diseased” state, particularly in the context of T1D, where these cells were more prevalent compared with HCs (Figure 5A). Slingshot identified 2 lineages: one progressing from PT-3, -2, -5, and -4 and the other from PT-3, -2, and -1 (Figure 5D). For this study, we focused on the first lineage (PT-3, -2, -5, -4) due to its relevance to our research objectives. Spearman’s correlations were calculated between the pseudotime values and clinical metrics, including HbA1c, BMI, M-value, GBM thickness, and average cortical k_2_, to assess the relationship between cell state progression and disease severity indicators. Figure 9 illustrates the pseudotime analysis concept.

### Statistics.

Participant characteristics were summarized as count and percentages, mean and SD, or median and IQR based on visual inspection of histograms for distribution. The HC and T1D groups were compared using χ^2^ tests or Fisher’s exact tests for categorical variables and using *t* tests and Mann-Whitney *U* tests for continuous variables. We conducted Spearman’s correlations between GBM thickness and cortical *k_2_* with tissue metabolites, limited to the T1D group. Additionally, we examined Spearman’s correlations between GBM thickness and M-value with PET parameters, involving both T1D patients and HCs.

We conducted linear regression analyses to assess associations in the correlations, adjusting for clinically relevant covariates. Covariates included age, sex, and HbA1c for assessing whole body insulin sensitivity as a predictor for cortical and medullary *k_2_*. Steady-state insulin concentrations and body fat percentage were considered to compare whole body insulin sensitivity between groups, baseline NEFA concentrations for comparing NEFA suppression between groups, and kidney perfusion for comparing furosemide-suppressible consumption between groups.

Spatial metabolomic tissue data were visually inspected using histograms. Duplicate measurements from each tissue sample were averaged and analyzed using square root transformed regression in consideration of right-skewed data. *P* < 0.05 was considered statistically significant. Analyses were performed using Python, v.3.9.6, and R, v.4.2.2 (The R Foundation for Statistical Computing).

### Study approval.

The study was approved by the Colorado Multiple Institutional Review Board (COMIRB) with approval number #19-1282. Participants provided written informed consent to participate in the main study. The study also included 2 optional procedures: a ^11^C-acetate kidney PET/CT scan and a research kidney biopsy, which included separate consent forms. Those who opted to undergo the optional kidney biopsy were additionally consented for that procedure.

### Data availability.

The data underlying the tables and figures of the manuscript are available in the Supporting Data Values file. The scRNA count data are available through the NCBI’s Gene Expression Omnibus database (GEO GSE279086).

## Author contributions

YJC and PB wrote the first draft of the manuscript. PB, YJC, CB, SG, AM, PN, LPL, PP, LD, KT, KJN, MF, VNS, TV, JK, and JKSB performed the research procedures, researched data, and contributed to discussion and reviewed/edited the manuscript. GR, DPB, and BBC performed the C-11 acetate PET postprocessing and analyses. PL performed the research kidney biopsies. LPL and PP performed the multiparametric MRI postprocessing and analyses. K Sharma, YY, IMT, GZ, JBH, MAV, DSD, BKG, and AR performed spatial metabolomics and light and electron microscopy morphometrics. MK, K Sharma, JAS, ASN, VN, PJM, FA, EAO, FCB, and VGP performed the scRNA-Seq, postprocessing, and analyses. IHDB, DZC, DHVR, JAG, RGN, PJS, and MK contributed to discussion and reviewed/edited the manuscript. PB designed the study. YJC, K Street, TV, and LP performed the analyses and contributed to discussion and reviewed/edited the manuscript. PB, LP, and YJC are the guarantors of this work and, as such, had full access to all the data in the study and take responsibility for the integrity of the data and the accuracy of the data analysis.

## Supplementary Material

Supplemental data

ICMJE disclosure forms

Supporting data values

## Figures and Tables

**Table 5:**
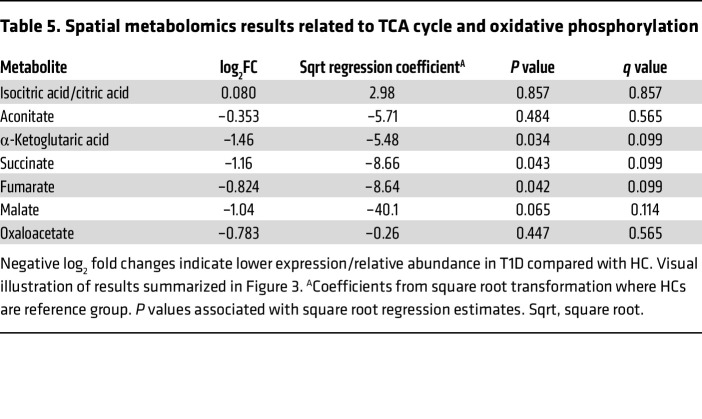
Spatial metabolomics results related to TCA cycle and oxidative phosphorylation

**Table 4:**
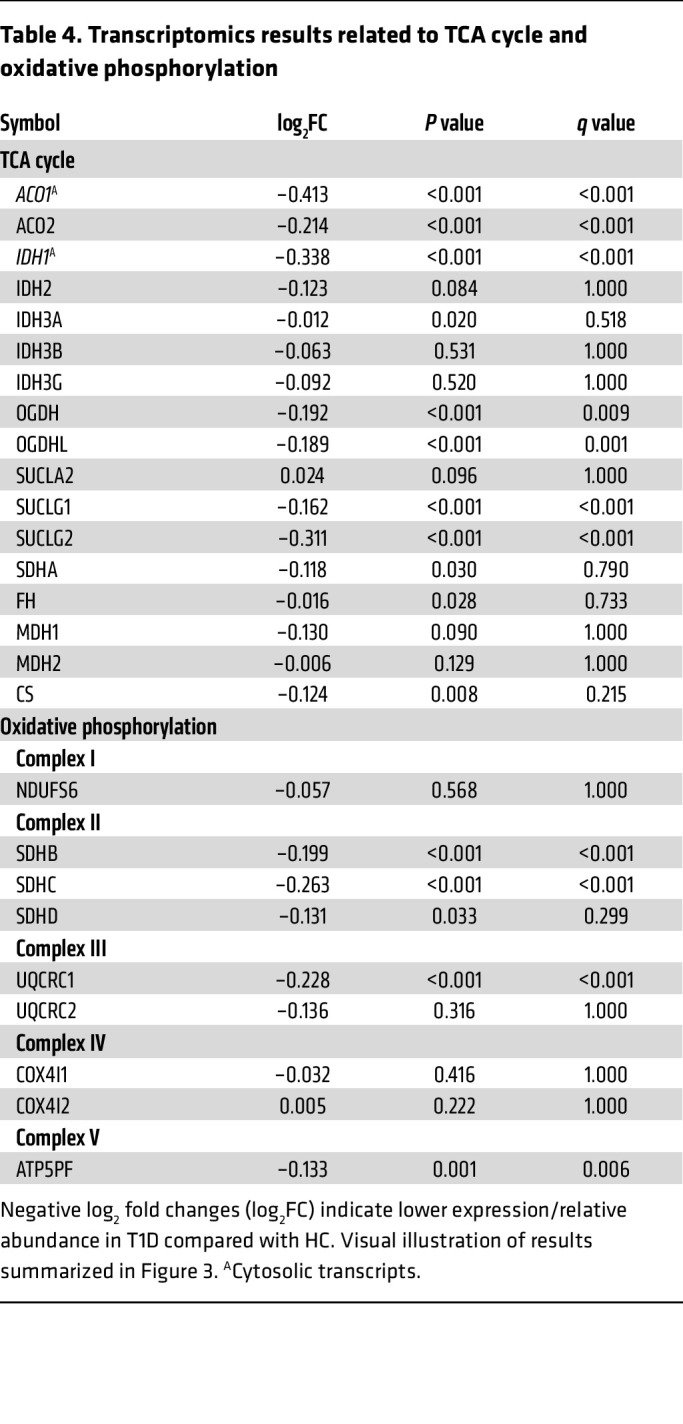
Transcriptomics results related to TCA cycle and oxidative phosphorylation

**Table 3:**
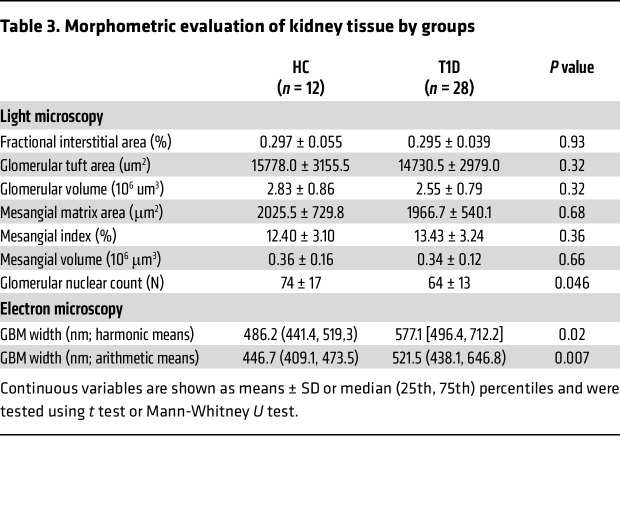
Morphometric evaluation of kidney tissue by groups

**Table 2:**
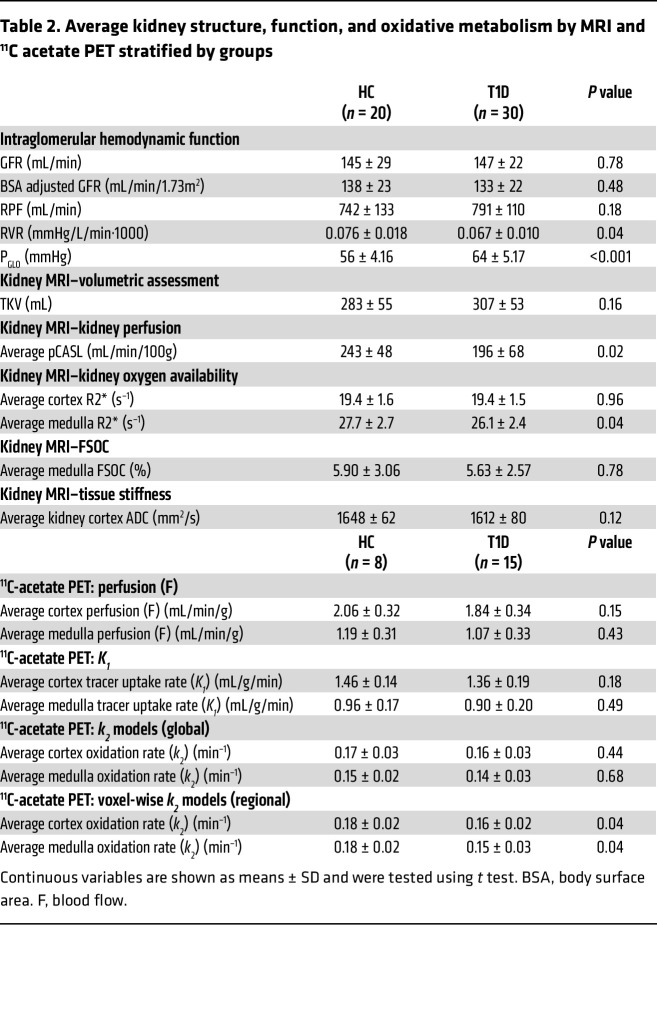
Average kidney structure, function, and oxidative metabolism by MRI and ^11^C acetate PET stratified by groups

**Table 1:**
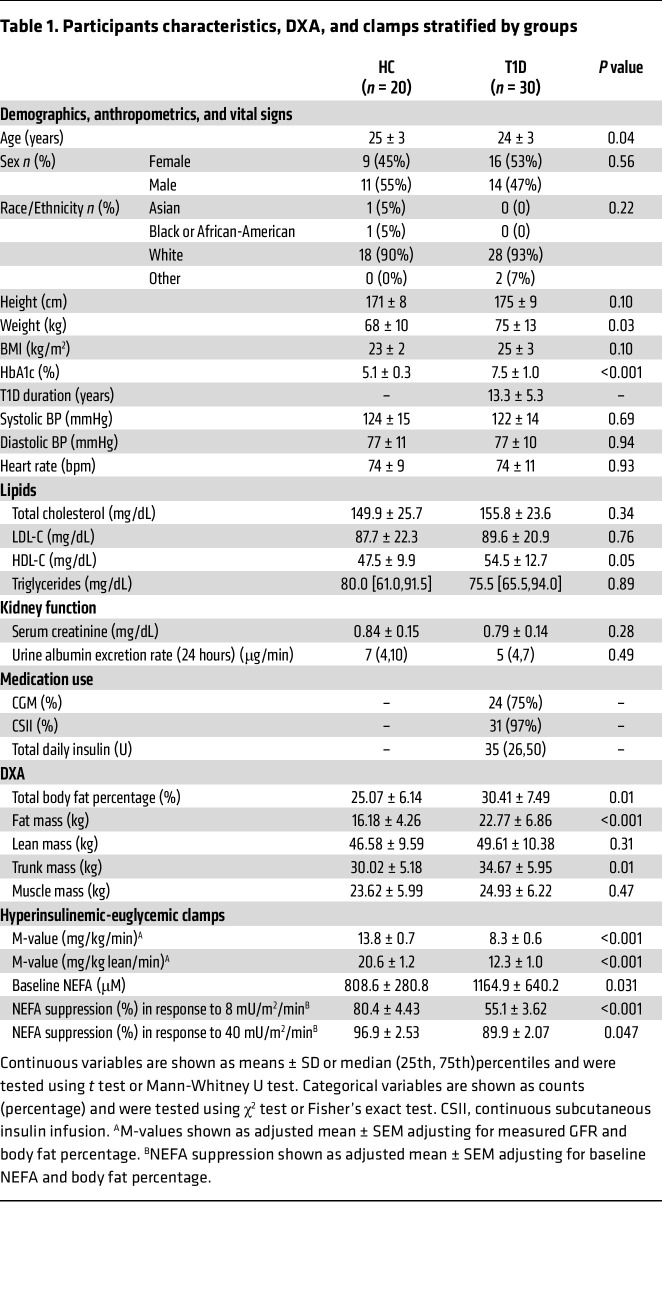
Participants characteristics, DXA, and clamps stratified by groups

## Version 1 10/22/2024

In-Press Preview

## Version 2 12/16/2024

Electronic publication

## References

[1] Rapaport R, Sills IN (1994). Implications of the DCCT for children and adolescents with IDDM. N J Med.

[2] Purnell JQ (1998). Effect of excessive weight gain with intensive therapy of type 1 diabetes on lipid levels and blood pressure: results from the DCCT. Diabetes Control and Complications Trial. JAMA.

[3] Purnell JQ (2017). Impact of excessive weight gain on cardiovascular outcomes in type 1 diabetes: results from the diabetes control and complications trial/epidemiology of diabetes interventions and complications (DCCT/EDIC) Study. Diabetes Care.

[4] de Boer IH (2007). Central obesity, incident microalbuminuria, and change in creatinine clearance in the epidemiology of diabetes interventions and complications study. J Am Soc Nephrol.

[5] Gubitosi-Klug R (2021). Associations of microvascular complications with the risk of cardiovascular disease in type 1 diabetes. Diabetes Care.

[6] Tuttle KR (2014). Diabetic kidney disease: a report from an ADA Consensus Conference. Diabetes Care.

[7] Lake BB (2023). An atlas of healthy and injured cell states and niches in the human kidney. Nature.

[8] de Boer IH (2014). Renal outcomes in patients with type 1 diabetes and macroalbuminuria. J Am Soc Nephrol.

[9] de Boer IH (2011). Long-term renal outcomes of patients with type 1 diabetes mellitus and microalbuminuria: an analysis of the Diabetes Control and Complications Trial/Epidemiology of Diabetes Interventions and Complications cohort. Arch Intern Med.

[10] Afkarian M (2016). Clinical manifestations of kidney disease among US adults with diabetes, 1988-2014. JAMA.

[11] de Boer IH, Group DER (2014). Kidney disease and related findings in the diabetes control and complications trial/epidemiology of diabetes interventions and complications study. Diabetes Care.

[12] de Boer IH (2011). Temporal trends in the prevalence of diabetic kidney disease in the United States. JAMA.

[13] Penno G (2017). Evidence for two distinct phenotypes of chronic kidney disease in individuals with type 1 diabetes mellitus. Diabetologia.

[14] Molitch ME (2010). Development and progression of renal insufficiency with and without albuminuria in adults with type 1 diabetes in the diabetes control and complications trial and the epidemiology of diabetes interventions and complications study. Diabetes Care.

[15] Perkins BA (2007). Microalbuminuria and the risk for early progressive renal function decline in type 1 diabetes. J Am Soc Nephrol.

[16] DCCT/EDIC Research Group (2011). Intensive diabetes therapy and glomerular filtration rate in type 1 diabetes. N Engl J Med.

[17] DCCT/EDIC research group. (2014). Effect of intensive diabetes treatment on albuminuria in type 1 diabetes: long-term follow-up of the Diabetes Control and Complications Trial and Epidemiology of Diabetes Interventions and Complications study. Lancet Diabetes Endocrinol.

[18] Amiel SA (1986). Impaired insulin action in puberty. A contributing factor to poor glycemic control in adolescents with diabetes. N Engl J Med.

[19] Cook JS (1993). Effects of maturational stage on insulin sensitivity during puberty. J Clin Endocrinol Metab.

[20] Roemmich JN (2002). Pubertal alterations in growth and body composition. VI. Pubertal insulin resistance: relation to adiposity, body fat distribution and hormone release. Int J Obes Relat Metab Disord.

[21] Travers SH (1995). Gender and Tanner stage differences in body composition and insulin sensitivity in early pubertal children. J Clin Endocrinol Metab.

[22] Packer M (2020). Role of deranged energy deprivation signaling in the pathogenesis of cardiac and renal disease in states of perceived nutrient overabundance. Circulation.

[23] Afshinnia F (2019). Increased lipogenesis and impaired β-oxidation predict type 2 diabetic kidney disease progression in American Indians. JCI Insight.

[24] Rebelos E (2019). Renal hemodynamics and fatty acid uptake: effects of obesity and weight loss. Am J Physiol Endocrinol Metab.

[25] Henry RR (1991). Dose-response characteristics of impaired glucose oxidation in non-insulin-dependent diabetes mellitus. Am J Physiol.

[26] Liu LL (2010). Prevalence of overweight and obesity in youth with diabetes in USA: the SEARCH for Diabetes in Youth study. Pediatr Diabetes.

[27] Libman IM (2003). Changing prevalence of overweight children and adolescents at onset of insulin-treated diabetes. Diabetes Care.

[28] Lipman TH (2013). Increasing incidence of type 1 diabetes in youth: twenty years of the Philadelphia Pediatric Diabetes Registry. Diabetes Care.

[29] Soltoff SP (1986). ATP and the regulation of renal cell function. Annu Rev Physiol.

[30] Hesp AC (2020). The role of renal hypoxia in the pathogenesis of diabetic kidney disease: a promising target for newer renoprotective agents including SGLT2 inhibitors?. Kidney Int.

[31] Cohen JJ (1979). Is the function of the renal papilla coupled exclusively to an anaerobic pattern of metabolism?. Am J Physiol.

[32] Umino H (2018). High basolateral glucose increases sodium-glucose cotransporter 2 and reduces sirtuin-1 in renal tubules through glucose transporter-2 detection. Sci Rep.

[33] Sakai S (2019). Proximal tubule autophagy differs in type 1 and 2 diabetes. J Am Soc Nephrol.

[34] Krzysiak TC (2018). An insulin-responsive sensor in the SIRT1 disordered region binds DBC1 and PACS-2 to control enzyme activity. Mol Cell.

[35] Sasaki M (2017). Dual regulation of gluconeogenesis by insulin and glucose in the proximal tubules of the kidney. Diabetes.

[36] Packer M (2020). Role of impaired nutrient and oxygen deprivation signaling and deficient autophagic flux in diabetic CKD development: implications for understanding the effects of sodium-glucose cotransporter 2-inhibitors. J Am Soc Nephrol.

[37] Tomita I (2020). SGLT2 inhibition mediates protection from diabetic kidney disease by promoting ketone body-induced mTORC1 inhibition. Cell Metab.

[38] Kogot-Levin A (2020). Proximal tubule mTORC1 is a central player in the pathophysiology of diabetic nephropathy and its correction by SGLT2 inhibitors. Cell Rep.

[39] Hallan S (2017). Metabolomics and gene expression analysis reveal down-regulation of the Citric Acid (TCA) cycle in non-diabetic CKD patients. EBioMedicine.

[40] Liu H (2021). Changes in plasma and urine metabolites associated with empagliflozin in patients with type 1 diabetes. Diabetes Obes Metab.

[41] Sridhar VS (2024). Chronic kidney disease in type 1 diabetes: translation of novel type 2 diabetes therapeutics to individuals with type 1 diabetes. Diabetologia.

[42] Kugathasan L (2024). Minireview: Understanding and targeting inflammatory, hemodynamic and injury markers for cardiorenal protection in type 1 diabetes. Metabolism.

[43] Heerspink HJ (2023). People with type 1 diabetes and chronic kidney disease urgently need new therapies: a call for action. Lancet Diabetes Endocrinol.

[44] Sharma K (2013). Metabolomics reveals signature of mitochondrial dysfunction in diabetic kidney disease. J Am Soc Nephrol.

[45] Qi H (2017). Glomerular endothelial mitochondrial dysfunction is essential and characteristic of diabetic kidney disease susceptibility. Diabetes.

[46] Zhang G (2018). The warburg effect in diabetic kidney disease. Semin Nephrol.

[47] Saulnier PJ (2021). Intraglomerular dysfunction predicts kidney failure in type 2 diabetes. Diabetes.

[48] Horton WB, Barrett EJ (2021). Microvascular dysfunction in diabetes mellitus and cardiometabolic disease. Endocr Rev.

[49] Alicic RZ (2017). Diabetic kidney disease: challenges, progress, and possibilities. Clin J Am Soc Nephrol.

[50] O’Connor PM (2006). Renal oxygen delivery: matching delivery to metabolic demand. Clin Exp Pharmacol Physiol.

[51] Brezis M, Rosen S (1995). Hypoxia of the renal medulla — its implications for disease. N Engl J Med.

[52] Edwards A, Kurtcuoglu V (2022). Renal blood flow and oxygenation. Pflugers Arch.

[53] Bjornstad P (2023). Insulin secretion, sensitivity, and kidney function in young individuals with type 2 diabetes. Diabetes Care.

[54] Lytvyn Y (2022). Renal and vascular effects of combined SGLT2 and angiotensin-converting enzyme inhibition. Circulation.

[55] Bangstad HJ (1994). Improvement of blood glucose control in IDDM patients retards the progression of morphological changes in early diabetic nephropathy. Diabetologia.

[56] Coughlan MT (2016). Mapping time-course mitochondrial adaptations in the kidney in experimental diabetes. Clin Sci (Lond).

[57] DeFronzo RA (1979). Glucose clamp technique: a method for quantifying insulin secretion and resistance. Am J Physiol.

[58] Nadeau KJ (2009). Insulin resistance in adolescents with type 2 diabetes is associated with impaired exercise capacity. J Clin Endocrinol Metab.

[59] Kamel EG (1999). Measurement of abdominal fat by magnetic resonance imaging, dual-energy X-ray absorptiometry and anthropometry in non-obese men and women. Int J Obes Relat Metab Disord.

[60] McCarthy C (2023). Total and regional appendicular skeletal muscle mass prediction from dual-energy X-ray absorptiometry body composition models. Sci Rep.

[61] Battilana C (1991). PAH extraction and estimation of plasma flow in diseased human kidneys. Am J Physiol.

[62] Corrigan G (1999). PAH extraction and estimation of plasma flow in human postischemic acute renal failure. Am J Physiol.

[63] Smith HW (1938). The measurement of the tubular excretory mass, effective blood flow and filtration rate in the normal human kidney. J Clin Invest.

[64] Looker HC (2019). Changes in albuminuria but not GFR are associated with early changes in kidney structure in type 2 diabetes. J Am Soc Nephrol.

[65] Melsom T (2019). Correlation between baseline GFR and subsequent change in GFR in Norwegian adults without diabetes and in Pima Indians. Am J Kidney Dis.

[66] Tanamas SK (2016). Long-term effect of losartan on kidney disease in American Indians with type 2 diabetes: a follow-up analysis of a Randomized Clinical Trial. Diabetes Care.

[67] Fufaa GD (2016). Structural predictors of loss of renal function in American Indians with type 2 diabetes. Clin J Am Soc Nephrol.

[68] Vinovskis C (2020). Relative hypoxia and early diabetic kidney disease in type 1 diabetes. Diabetes.

[69] Bjornstad P (2016). The Gomez’ equations and renal hemodynamic function in kidney disease research. Am J Phsyiol Renal Physiol.

[70] Cherney DZ (2010). Renal hyperfiltration is a determinant of endothelial function responses to cyclooxygenase 2 inhibition in type 1 diabetes. Diabetes Care.

[71] Skrtic M (2015). Glomerular haemodynamic profile of patients with Type 1 diabetes compared with healthy control subjects. Diabet Med.

[72] Prasad PV (2015). Multi-parametric evaluation of chronic kidney disease by MRI: a preliminary cross-sectional study. PLoS One.

[73] Juillard L (2007). Validation of renal oxidative metabolism measurement by positron-emission tomography. Hypertension.

[74] Normand G (2019). PET [^11^C]acetate is also a perfusion tracer for kidney evaluation purposes. Nucl Med Biol.

[75] Schaub JA (2022). Quantitative morphometrics reveals glomerular changes in patients with infrequent segmentally sclerosed glomeruli. J Clin Pathol.

[76] Hodgin JB (2015). Glomerular aging and focal global glomerulosclerosis: a podometric perspective. J Am Soc Nephrol.

[77] Harder JL (2019). Organoid single cell profiling identifies a transcriptional signature of glomerular disease. JCI Insight.

[78] Menon R (2018). Single-cell analysis of progenitor cell dynamics and lineage specification in the human fetal kidney. Development.

[79] Arazi A (2019). The immune cell landscape in kidneys of lupus nephritis patients. Nat Immunol.

[80] Menon R (2020). Single cell transcriptomics identifies focal segmental glomerulosclerosis remission endothelial biomarker. JCI Insight.

[81] Lake BB (2023). An atlas of healthy and injured cell states and niches in the human kidney. Nature.

[82] Sharma K (2023). Endogenous adenine mediates kidney injury in diabetic models and predicts diabetic kidney disease in patients. J Clin Invest.

[83] Belov ME (2017). Design and performance of a novel interface for combined matrix-assisted laser desorption ionization at elevated pressure and electrospray ionization with orbitrap mass spectrometry. Anal Chem.

[84] https://www.protocols.io/view/optical-image-collection-dm6gpr5x5vzp/v1.

[85] Palmer A (2017). FDR-controlled metabolite annotation for high-resolution imaging mass spectrometry. Nat Methods.

[86] Drummond K (2002). The early natural history of nephropathy in type 1 diabetes: II. Early renal structural changes in type 1 diabetes. Diabetes.

