# Supplementary material for: Attenuated kidney oxidative metabolism in young adults with type 1 diabetes
Source: J Clin Invest. 2024 Oct 22;134(24):e183984. doi: 10.1172/JCI183984 (PMC11645151; doi:10.1172/JCI183984)
Supplement: Supplemental data [file jci-134-183984-s159.pdf]

Figure S1 Heatmap of correlations between GBM thickness and M-value with PET parameters adjusting for sex, HbA1c, and age

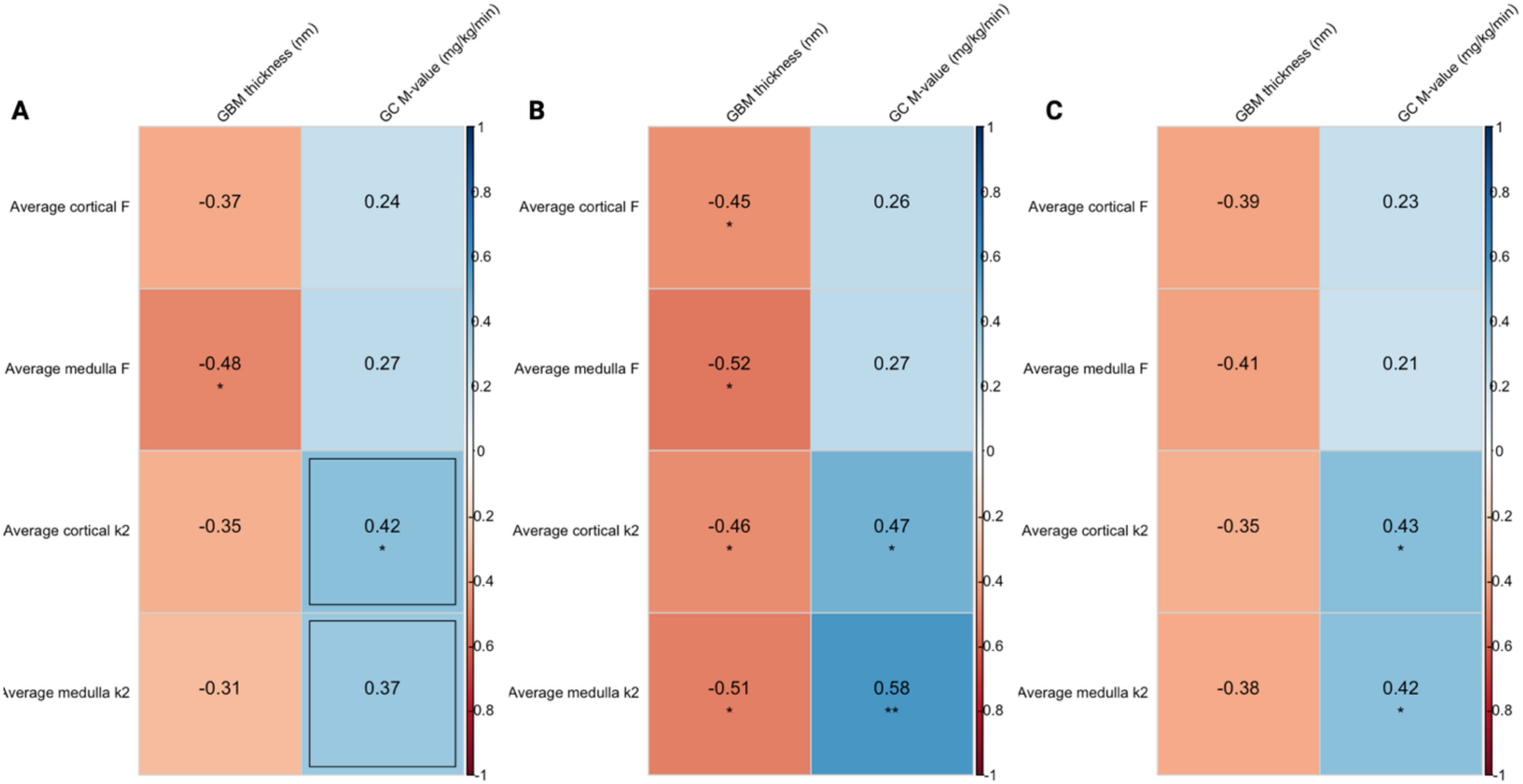

(A) Spearman correlation heatmap showing the correlation coefficients, represented by the numbers within each box. Stars indicate the significance level of each Spearman correlation. Boxes outlined in black highlight significant relationships ($p < 0.05$) as determined by regression analysis adjusting for sex. **(B)** Partial Spearman correlations adjusting for HbA1c. **(C)** Partial Spearman correlations adjusting for age.

Figure S2 UMAP of scRNAseq data from kidney biopsies obtained in participants with type 1 diabetes and healthy controls

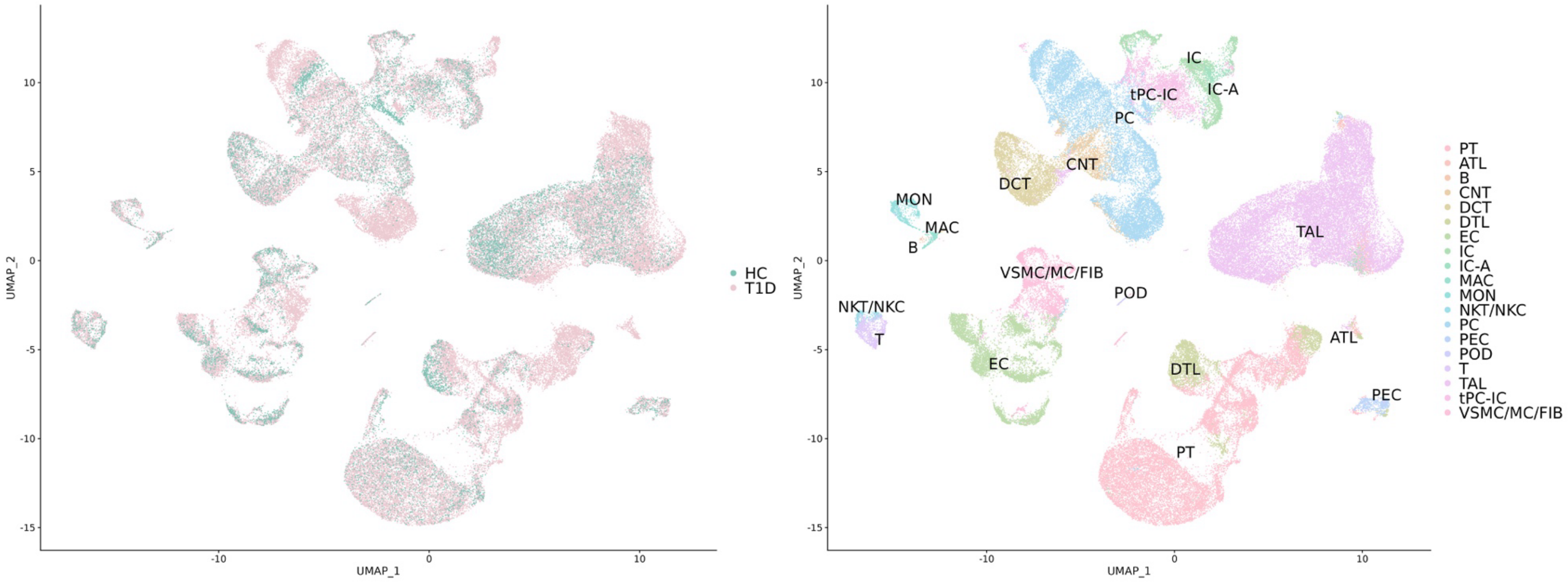

ScRNAseq cell type clusters are presented in UMAP. The UMAP on the left is colored by group (HC in blue and T1D in pink), and shows even distribution of cell types across groups. The UMAP on the right is colored and labeled by cell types.
Abbreviations: UMAP, uniform manifold approximation and projection; scRNAseq, single cell ribonucleic acid sequencing; HC, healthy control; T1D, type 1 diabetes; PT, proximal tubule; ATL, ascending thin limbs; B, B cells; CNT, connecting tubule; DCT, distal convoluted tubule; EC, endothelial cells; IC, intercalated cells; IC-A, intercalated cell type A; MAC, macrophage; MON, monocyte; NKT/NKC, natural T-cells/killer cells; PC, principal cells; PEC, parietal endothelial cells; POD, podocyte; T, T-cells; TAL, thick ascending limb; tPC-IC, transitioning principal and intercalated cells; VSMC/MC/FIB, vascular smooth muscle/mesangium/fibroblast.

Figure S3 Distribution of cell types in scRNAseq analysis

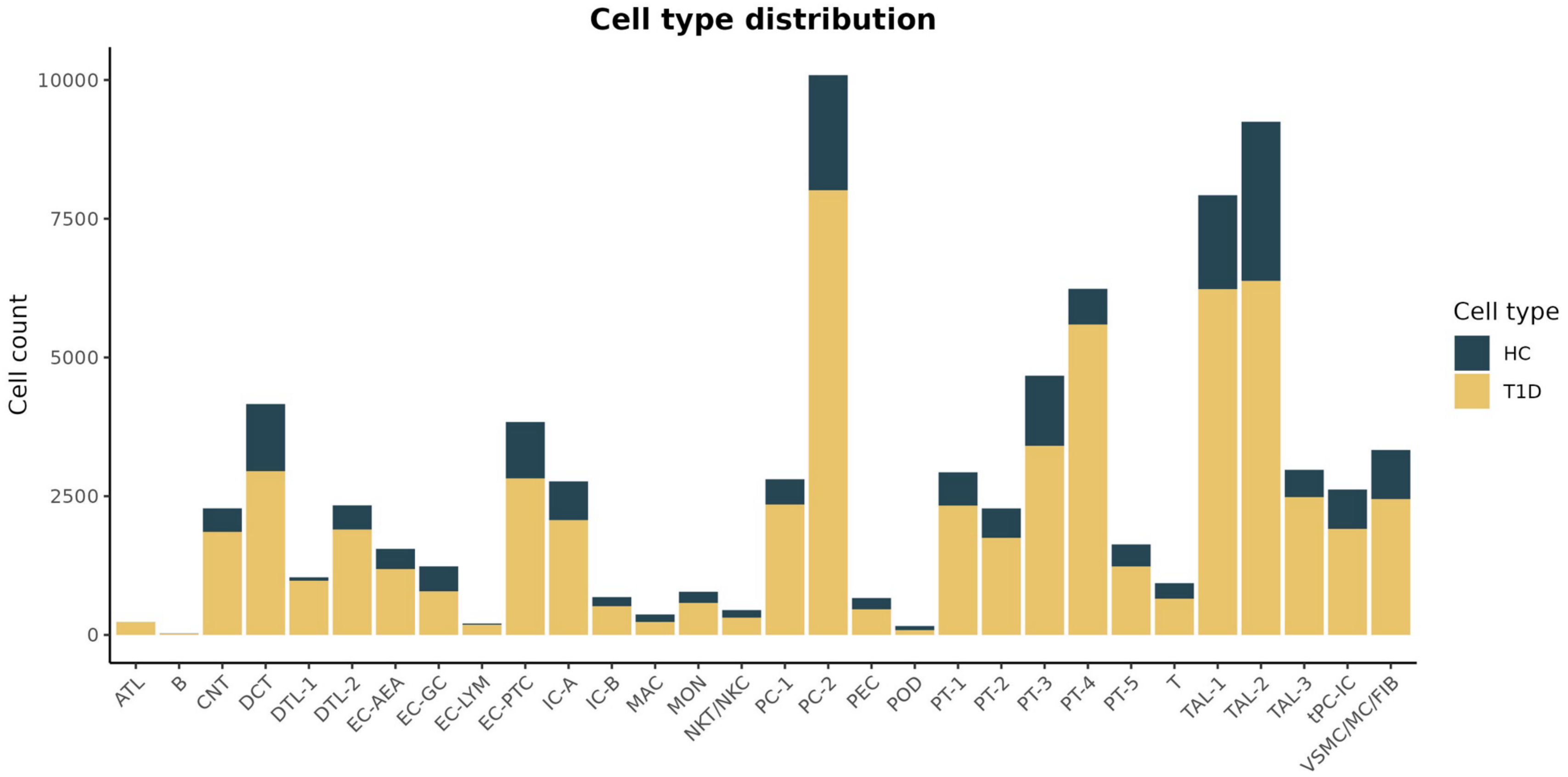

Distribution of cell types in scRNAseq data analysis and proportion of group type shown as proportions within each bar. PC, PT, and TAL cells were most abundant in dataset. Cells from T1D were more abundant than HC, greatly due to a higher sample size in T1D (N=28) than HC (N=12). Abbreviations: scRNAseq, single cell ribonucleic acid sequencing; HC, healthy control; T1D, type 1 diabetes; ATL, ascending thin limbs; B, B cells; CNT, connecting tubule; DCT, distal convoluted tubule; EC-AEA, endothelial cells-afferent/efferent arterioles; EC-GC, endothelial cells-glomerular endothelia; EC-LYM, endothelial cells-lymphatic endothelia; EC-PTC, endothelial cells-peritubular endothelia; IC-A, intercalated cells type A; IC-B, intercalated cell type B; MAC, macrophage; MON, monocyte; NKT/NKC, natural T-cells/killer cells; PC, principal cells; PEC, parietal endothelial cells; POD, podocyte; PT, proximal tubule; T, T-cells; TAL, thick ascending limb; tPC-IC, transitioning principal and intercalated cells; VSMC/MC/FIB, vascular smooth muscle/mesangium/fibroblast.

Figure S4 Bar plot of proximal tubular transcripts of enzymes catalyzing TCA cycle and oxidative phosphorylation

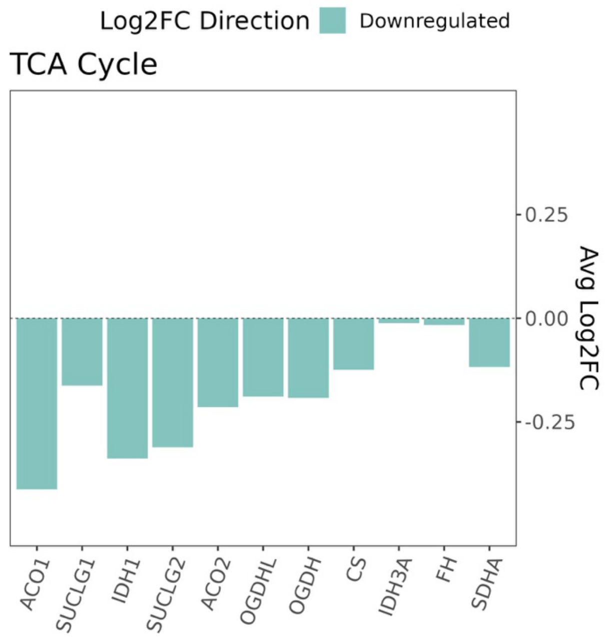

TCA cycle

	T1D	HC	Log2FC	p-value
ACO1	0.324	0.409	-0.413	<0.001
SUCLG1	0.603	0.679	-0.162	<0.001
IDH1	0.284	0.346	-0.338	<0.001
SUCLG2	0.320	0.375	-0.311	<0.001
ACO2	0.301	0.326	-0.214	<0.001
OGDHL	0.244	0.271	-0.189	<0.001
OGDH	0.260	0.282	-0.192	<0.001
CS	0.081	0.094	-0.124	0.0083
IDH3A	0.064	0.053	-0.012	0.0199
FH	0.121	0.106	-0.016	0.0282
SDHA	0.234	0.245	-0.118	0.0304

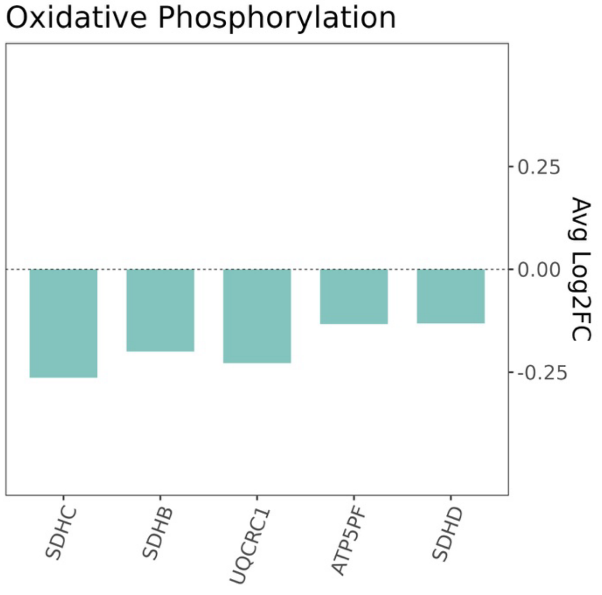

Oxidative Phosphorylation

	T1D	HC	Log2FC	p-value
SDHC	0.338	0.373	-0.263	<0.001
SDHB	0.266	0.301	-0.199	<0.001
UQCRC1	0.326	0.352	-0.228	<0.001
ATP5PF	0.641	0.633	-0.133	<0.001
SDHD	0.397	0.394	-0.131	0.0332

Average log2 fold change of transcripts involved in the TCA cycle and oxidative phosphorylation are plotted as bar plots, with blue bars indicating downregulation in T1D compared to HC. Analysis was limited to proximal tubular cells only.

Abbreviations: TCA cycle, tricarboxylic acid cycle; T1D, type 1 diabetes; HC, healthy control.

Figure S5 Bar plots of proximal tubular oxidative phosphorylation transcripts according to proximal tubular sub-cell types (PT1-5)

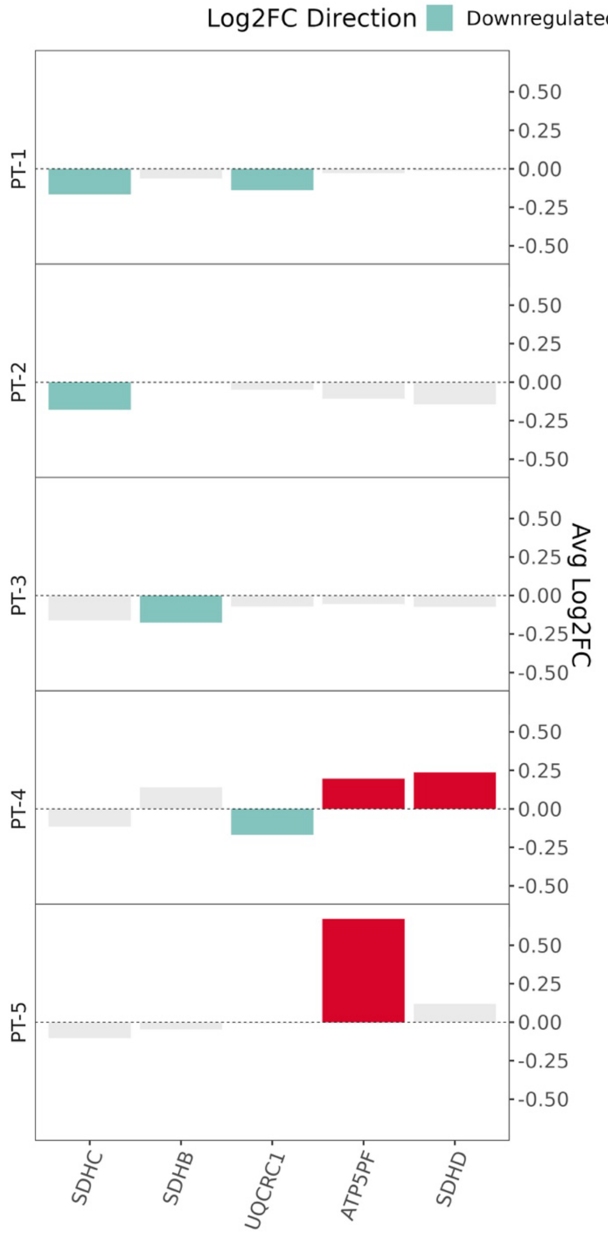

% Expressed in PT-1 Cells

	T1D	HC	Log2FC	p-value
SDHC	0.582	0.658	-0.166	<0.001
UQCRC1	0.630	0.700	-0.139	0.0025

% Expressed in PT-2 Cells

	T1D	HC	Log2FC	p-value
SDHC	0.402	0.430	-0.180	0.0391

% Expressed in PT-3 Cells

	T1D	HC	Log2FC	p-value
SDHB	0.245	0.279	-0.176	0.0051

% Expressed in PT-4 Cells

	T1D	HC	Log2FC	p-value
SDHD	0.331	0.280	0.237	0.0087
UQCRC1	0.253	0.291	-0.169	0.0167
ATP5PF	0.576	0.541	0.196	0.0371

% Expressed in PT-5 Cells

	T1D	HC	Log2FC	p-value
ATP5PF	0.144	0.075	0.671	<0.001

Average log2 fold change of transcripts involved in the oxidative phosphorylation are plotted as bar plots, with blue bars indicating downregulation and red bars indicating upregulation in T1D compared to HC. Not statistically significant (p>0.05) differential expression transcripts were plotted in grey. Analysis was limited to proximal tubular cells only, and results are presented in proximal tubular cell subtypes 1-5. Abbreviations: T1D, type 1 diabetes; HC, healthy control, PT, proximal tubular cells.

Figure S6 Spatial metabolic analysis reveals lower TCA cycle intermediates in regions of tubular pathology in participants with T1D compared to healthy controls.

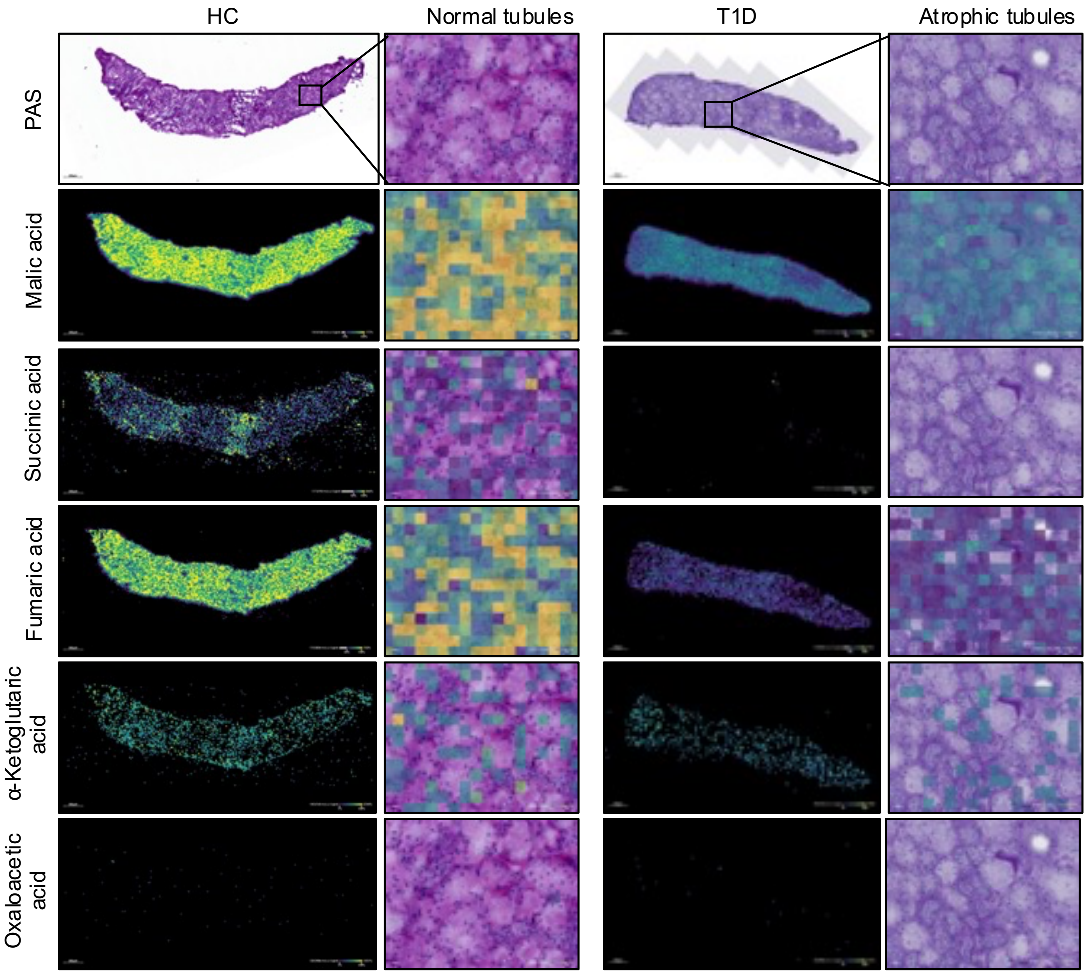

Kidney biopsies from individuals with type 1 diabetes (T1D) revealed discernible pathological alterations, notably atrophic tubules. Conversely, kidney biopsies obtained from healthy controls (HC) exhibited unremarkable and normal features. An example of atrophic tubules in T1D contrasted with normal tubules from HC is presented in this figure. Malic acid, succinic acid, fumaric acid, and α-ketoglutaric acid were localized to regions of normal tubules in the normal kidney. In diabetic kidneys, four metabolites were lower across the tissue section, especially in regions of atrophic tubules.

Figure S7 Semi-quantification of atrophic tubules in T1D vs HC.

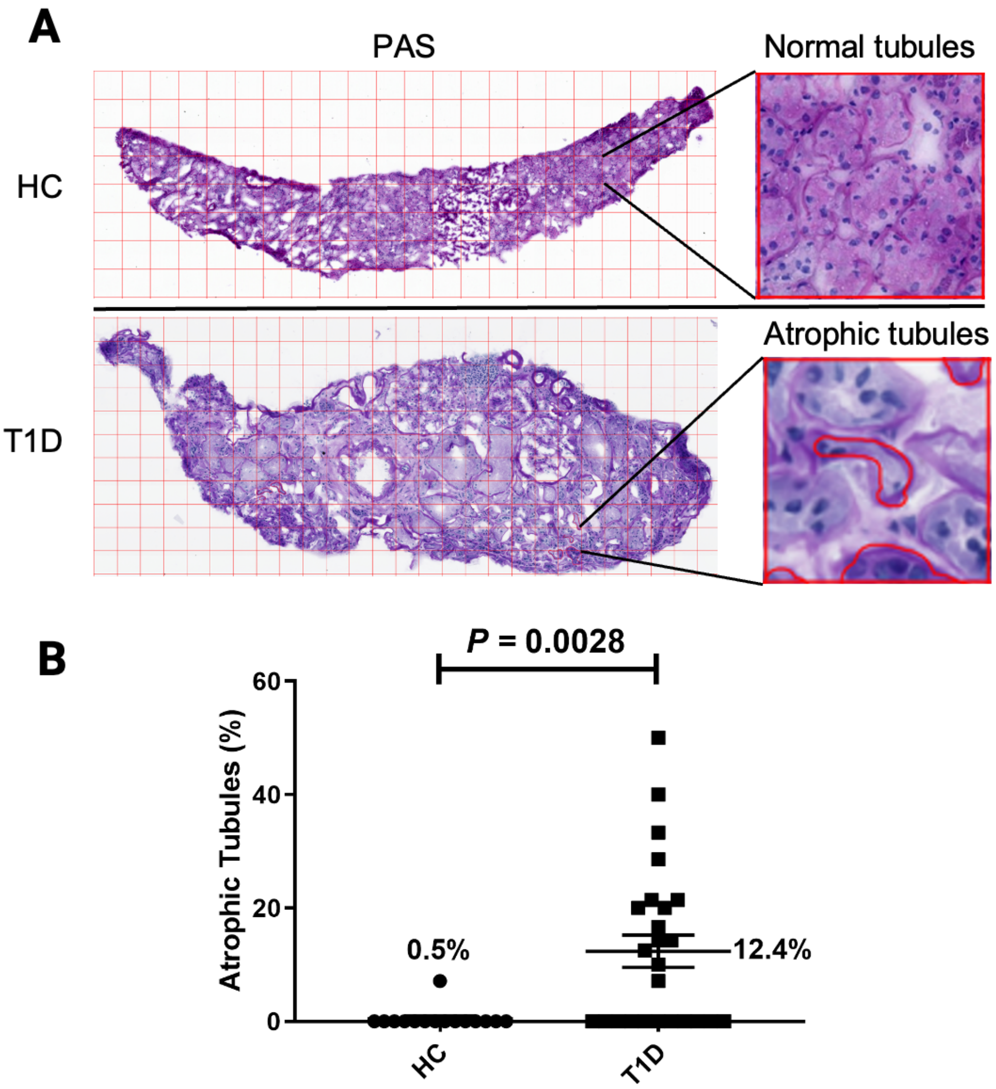

(A) Representative PAS images showed atrophic tubules (the area highlighted using red lines) in T1D and normal tubules in HC. **(B)** Semi-quantification of percentage of normal tubules and atrophic tubules in randomly selected regions of interests (ROIs) from each sample between T1D and HC. *t*-test was performed for the comparison of percentage of normal and atrophic tubules in healthy controls (HC: n=3; 5 ROIs per sample) and diabetic samples (T1D: n=5; 5 ROIs per sample).

Figure S8 Proportion of proximal tubular cell sub types

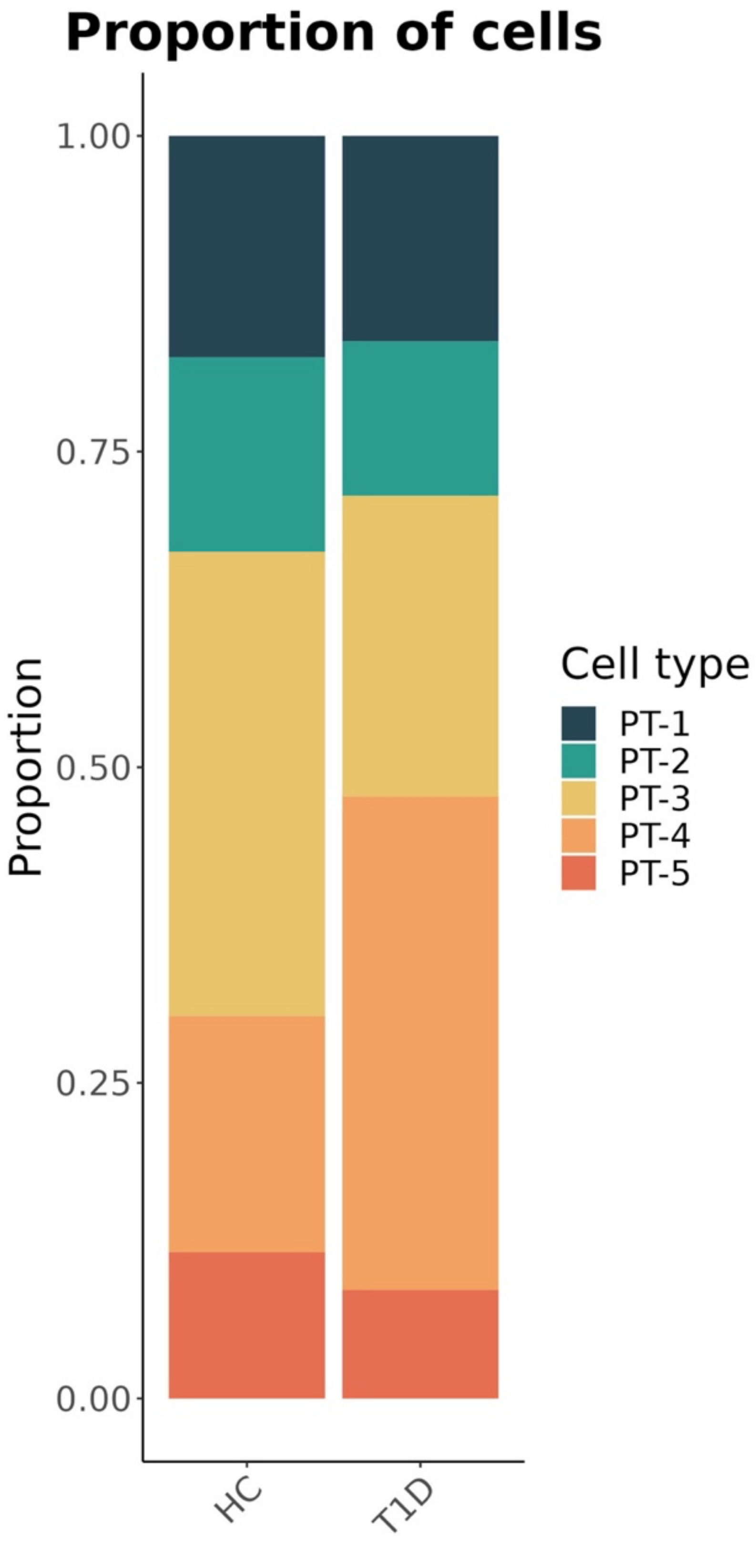

Proportion of PT cell sub types are presented as a bar plot. PT-3 cells were most abundant in HC and PT-4 cells were most abundant in T1D. PT-5 cells were least abundant in both HC and T1D.
Abbreviations: PT, proximal tubular; HC, healthy control; T1D, type 1 diabetes.

Figure S9 Dot plot of proximal tubular TCA cycle transcripts according to proximal tubular subtypes (PT1-5)

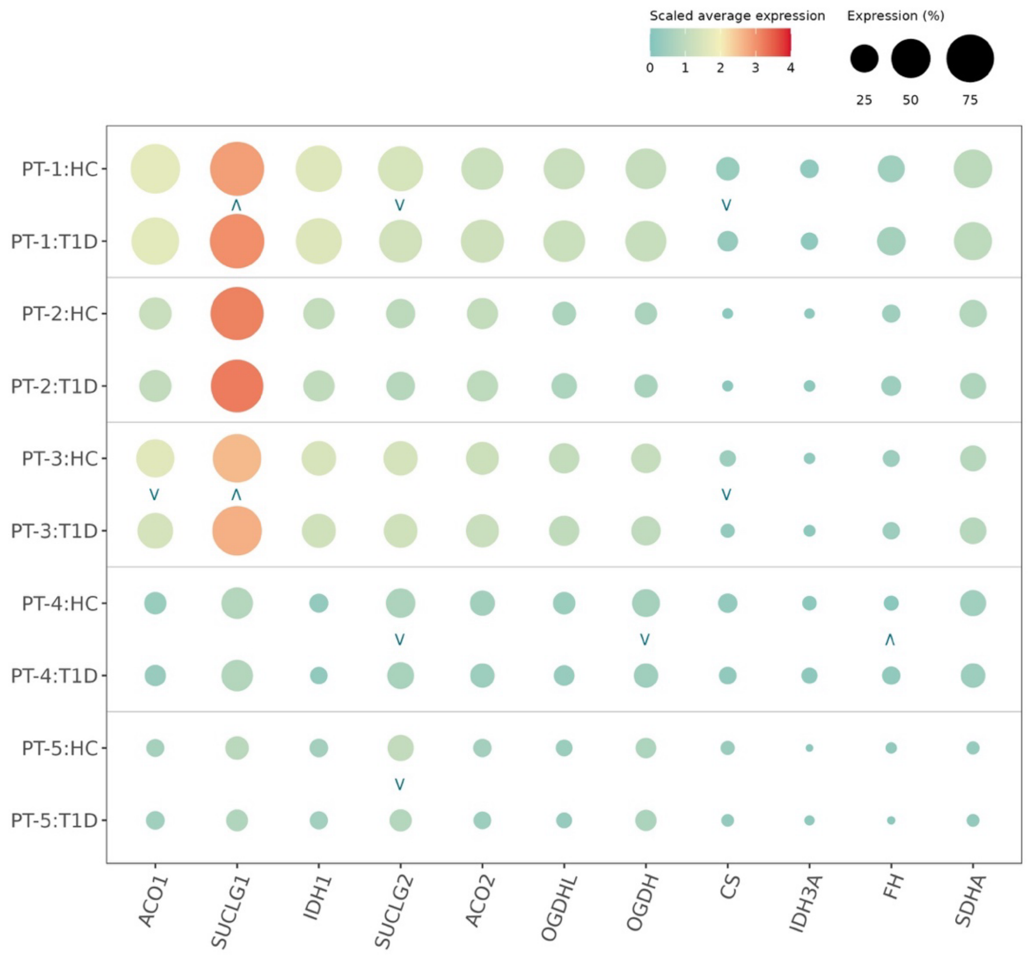

% Expressed in PT-1 Cells

	T1D	HC	Log2FC	p-value
SUCLG1	0.985	0.970	0.231	<0.001
SUCLG2	0.601	0.667	-0.164	0.0011
CS	0.130	0.171	-0.087	0.0123

% Expressed in PT-3 Cells

	T1D	HC	Log2FC	p-value
ACO1	0.412	0.474	-0.324	<0.001
SUCLG1	0.814	0.775	0.157	0.0058
CS	0.060	0.081	-0.180	0.0079

% Expressed in PT-4 Cells

	T1D	HC	Log2FC	p-value
OGDH	0.186	0.246	-0.130	<0.001
SUCLG2	0.228	0.277	-0.116	0.0024
FH	0.103	0.068	0.175	0.0050

% Expressed in PT-5 Cells

	T1D	HC	Log2FC	p-value
SUCLG2	0.153	0.218	-0.372	0.0018

Average expression of transcripts involved in the TCA cycle by group (HC vs. T1D) are shown in circles. Higher expression is presented by bigger circles and color closer to red. Greater than/less than signs between HC and T1D circles represent statistically significant differential expression. Abbreviations: PT, proximal tubular, T1D, type 1 diabetes; HC, healthy control.

Figure S10 Bar plots of proximal tubular TCA cycle transcripts according to proximal tubular sub-cell types (PT1-5)

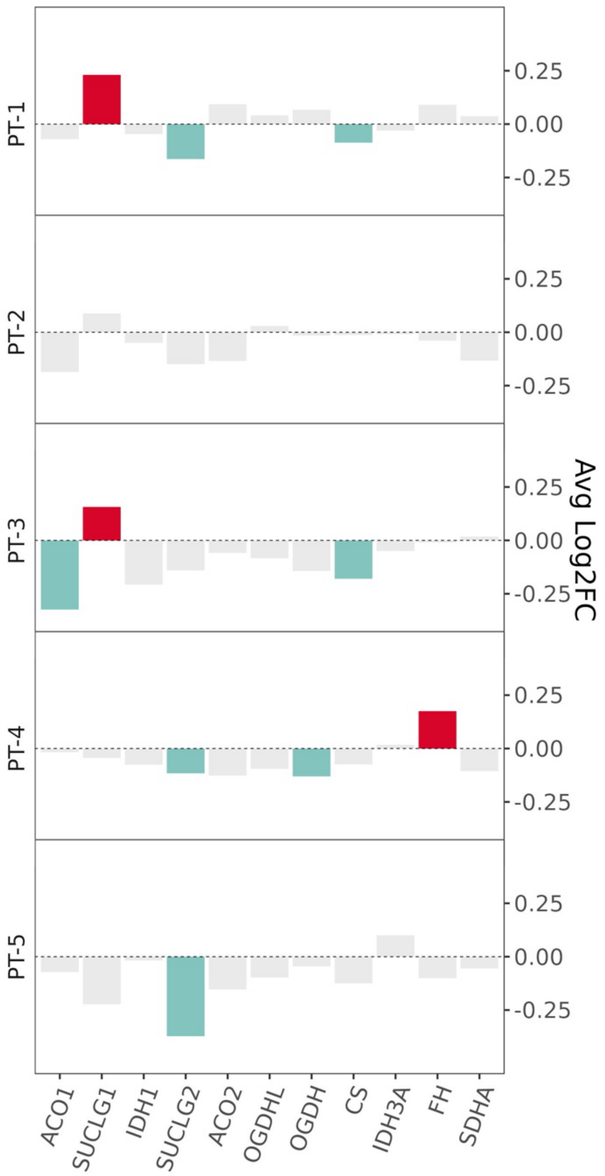

% Expressed in PT-1 Cells

	T1D	HC	Log2FC	p-value
SUCLG1	0.985	0.970	0.231	<0.001
SUCLG2	0.601	0.667	-0.164	0.0011
CS	0.130	0.171	-0.087	0.0123

% Expressed in PT-3 Cells

	T1D	HC	Log2FC	p-value
ACO1	0.412	0.474	-0.324	<0.001
SUCLG1	0.814	0.775	0.157	0.0058
CS	0.060	0.081	-0.180	0.0079

% Expressed in PT-4 Cells

	T1D	HC	Log2FC	p-value
OGDH	0.186	0.246	-0.130	<0.001
SUCLG2	0.228	0.277	-0.116	0.0024
FH	0.103	0.068	0.175	0.0050

% Expressed in PT-5 Cells

	T1D	HC	Log2FC	p-value
SUCLG2	0.153	0.218	-0.372	0.0018

Average log2 fold change of transcripts involved in the TCA cycle are plotted as bar plots, with blue bars indicating downregulation and red bars indicating upregulation in T1D compared to HC. Not statistically significant (p>0.05) differential expression transcripts were plotted in grey. Analysis was limited to proximal tubular cells only, and results are presented in proximal tubular cell subtypes 1-5.
Abbreviations: TCA cycle, tricarboxylic acid cycle; T1D, type 1 diabetes; HC, healthy control, PT, proximal tubular cells.

Figure S11 Raw PET signal in kidneys post injection by group

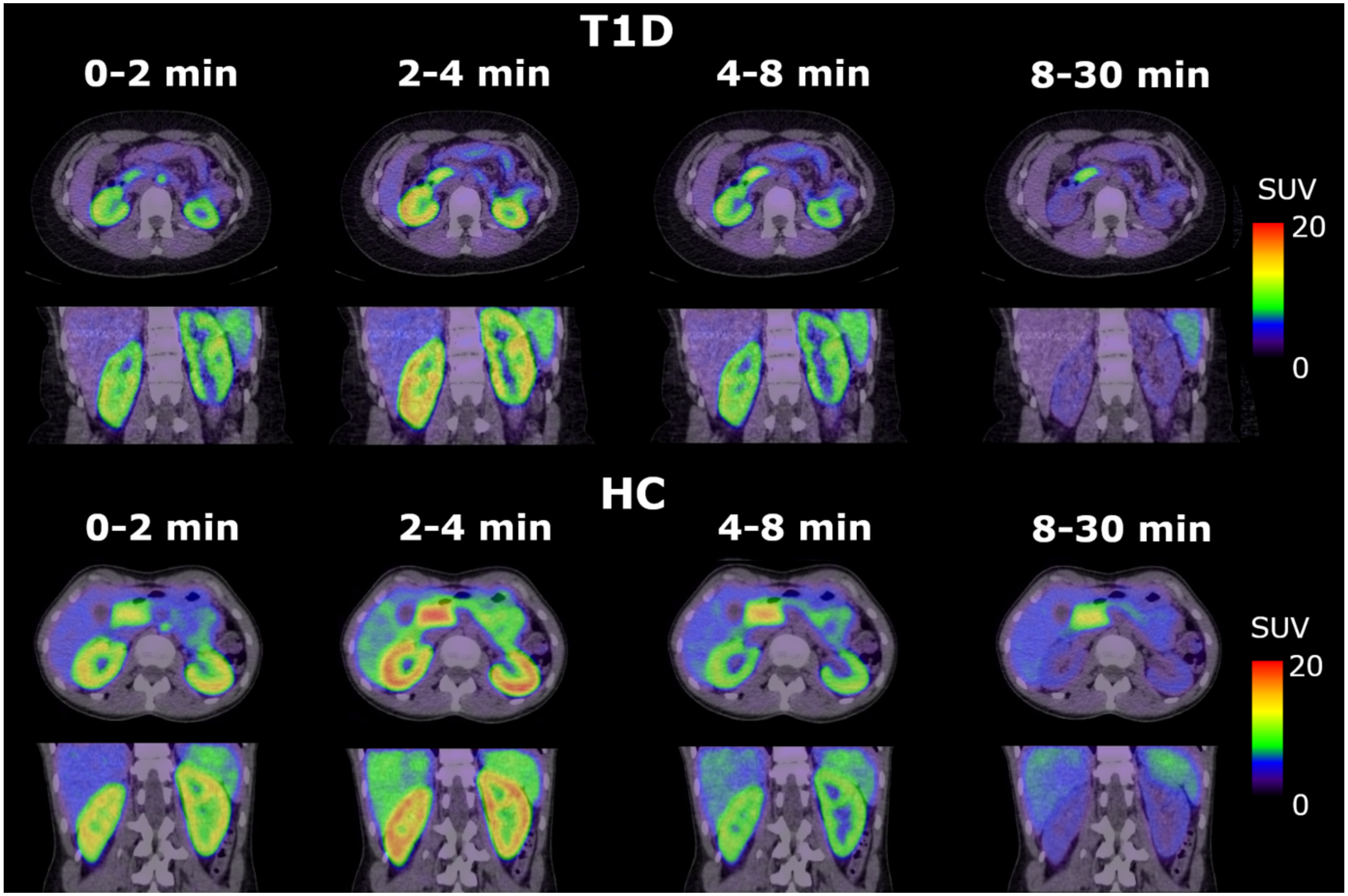

Axial and coronal views of the PET-CT image for a typical participant showing the average ^{11}C-acetate signal at different time points after radiotracer injection.
Abbreviations: T1D, type 1 diabetes, HC, healthy control; PET, position emission tomography; CT, computed tomography; SUV, standardized uptake value.

Figure S12 Example PET images

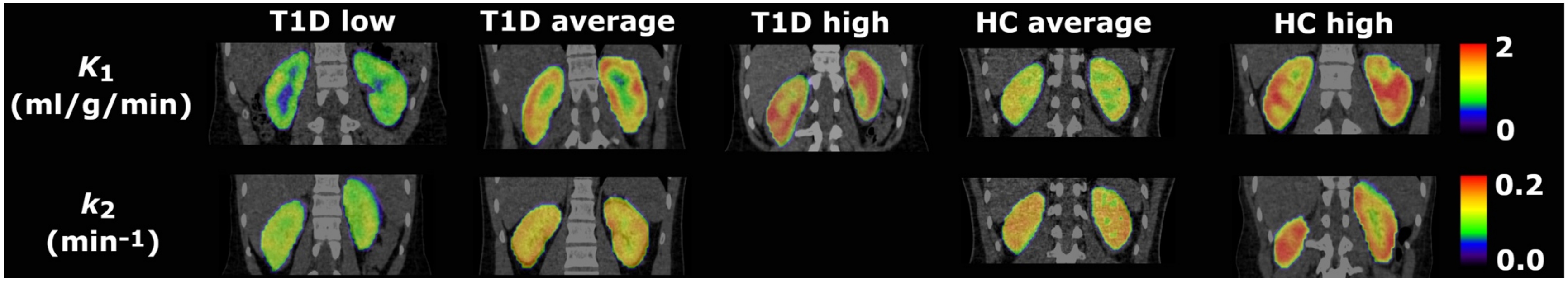

The ^{11}C-acetate turnover in the TCA cycle was estimated for each ROI using a one-tissue, two-compartment, pharmacokinetic model with parameters K_1 (tracer uptake), k_2 (tracer clearance, rate of CO2 production), and vb (blood volume fraction). Blood flow (F) was estimated using the K_1 values and assuming an extraction fraction of 0.52. A voxel-wise pharmacokinetic model provided parametric maps of K_1 and k_2 by fitting a one-tissue compartment model using linear ridge regression with spatial constraint.
